# Molecular profiling of fungal communities in moisture damaged buildings before and after remediation - a comparison of culture-dependent and culture-independent methods

**DOI:** 10.1186/1471-2180-11-235

**Published:** 2011-10-21

**Authors:** Miia Pitkäranta, Teija Meklin, Anne Hyvärinen, Aino Nevalainen, Lars Paulin, Petri Auvinen, Ulla Lignell, Helena Rintala

**Affiliations:** 1 Institute of Biotechnology, University of Helsinki, Viikinkaari 4, 00790 Helsinki, Finland; 2 Department of Environmental Health, National Institute for Health and Welfare, Neulaniementie 4, 70701 Kuopio, Finland; 3 Mikrobioni Inc., P.O. Box 1188, 70211 Kuopio, Finland

## Abstract

**Background:**

Indoor microbial contamination due to excess moisture is an important contributor to human illness in both residential and occupational settings. However, the census of microorganisms in the indoor environment is limited by the use of selective, culture-based detection techniques. By using clone library sequencing of full-length internal transcribed spacer region combined with quantitative polymerase chain reaction (qPCR) for 69 fungal species or assay groups and cultivation, we have been able to generate a more comprehensive description of the total indoor mycoflora. Using this suite of methods, we assessed the impact of moisture damage on the fungal community composition of settled dust and building material samples (n = 8 and 16, correspondingly). Water-damaged buildings (n = 2) were examined pre- and post- remediation, and compared with undamaged reference buildings (n = 2).

**Results:**

Culture-dependent and independent methods were consistent in the dominant fungal taxa in dust, but sequencing revealed a five to ten times higher diversity at the genus level than culture or qPCR. Previously unknown, verified fungal phylotypes were detected in dust, accounting for 12% of all diversity. Fungal diversity, especially within classes Dothideomycetes and Agaricomycetes tended to be higher in the water damaged buildings. Fungal phylotypes detected in building materials were present in dust samples, but their proportion of total fungi was similar for damaged and reference buildings. The quantitative correlation between clone library phylotype frequencies and qPCR counts was moderate (r = 0.59, p < 0.01).

**Conclusions:**

We examined a small number of target buildings and found indications of elevated fungal diversity associated with water damage. Some of the fungi in dust were attributable to building growth, but more information on the material-associated communities is needed in order to understand the dynamics of microbial communities between building structures and dust. The sequencing-based method proved indispensable for describing the true fungal diversity in indoor environments. However, making conclusions concerning the effect of building conditions on building mycobiota using this methodology was complicated by the wide natural diversity in the dust samples, the incomplete knowledge of material-associated fungi fungi and the semiquantitative nature of sequencing based methods.

## Background

Dampness or mold in buildings are positively associated with several allergic and respiratory effects [1]. Based on a meta-analysis of relevant literature, a 30-50% increase in variety of respiratory and asthma-related health outcomes was summarized by Fisk et al. [2]. It has also been estimated that 21% (4.6 million cases) of total asthma cases in the United States may be attributable to residential dampness and mold [3].

Due to the strong epidemiological association between observed dampness or mold and adverse health effects, it is hypothesized that excessive microbial proliferation in building materials manifests itself as increased or altered levels of microbe-derived compounds in the indoor air, which individually or in combination reach sufficient levels to affect human health. The elimination of growth by remediation is intended to normalize these levels, usually resulting in decreased symptoms [4-10]. However, alleviation is not always seen, especially if remediation has been partial [5,11,12]. At present, the agents that contribute to the development of the reported building-related health effects are still only partially understood, and no internationally accepted guidelines are available for monitoring the success of mold remediation [13]. This is due largely to the complex and compound nature of indoor exposures and the varying extent of population susceptibility, further complicated by traditional methodological deficiencies in the identification and enumeration of biological agents.

Fungi are major colonizers and degraders of building materials; they possess vast bioactive potential, and have the capacity to spread spores and smaller fragments from the site of proliferation to the surrounding air. The capacity to induce symptoms in the non-sensitized population at concentrations typical of indoor environments depends on species-specific traits, such as allergenicity, pathogenicity and mycotoxin production. Thus, the accurate identification of microbes is a prerequisite for the assessment of their potential health effects [14,15].

The present knowledge of indoor fungi relies on a long history of cultivation and direct microscopy, yet the use of these methods is known to bias the qualitative and quantitative community description [13,16,17]. Recently, quantitative PCR (qPCR) has been used for studying the levels of individual indoor mold species and assay groups [18-20], but few studies have thus far explored the total indoor mycobiota using DNA-based universal community characterization methods like ribosomal DNA amplicon sequencing or metagenome analysis [21-24]. Very little is known about the effect of building characteristics on the total fungal assemblages. A recent study by Amend et al. [21] suggested that indoor fungal communities are not significantly shaped by building-specific factors like building function, ventilation system or building materials, but instead global factors like geographic location and climate are more important. Unfortunately, the presence of water damage in buildings was not included among the studied factors, even though excess water is known to be the most significant individual factor associated with elevated viable fungal counts indoors [25,26].

The aim of the present study was to assess the fungal communities in moisture-damaged, renovated and non-damaged buildings using culture-based and culture-independent methods. Contaminated building materials collected from the subject buildings were analysed to determine if contaminants originating from these materials were likely to contribute to the fungal communities in the dust. In addition, we investigated the similarity of the fungal community profile revealed by sequencing, culture and a relatively large selection of targeted qPCR assays.

## Results

### Fungal diversity and comparison of methods

#### Fungi in dust samples

**Figure 1:**
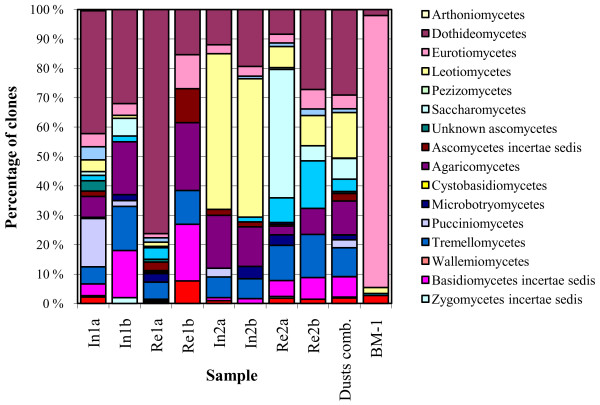
**Relative abundances of clones affiliated to fungal classes in the studied dust and building material samples**. Sample name abbreviations: In: index building Re: reference building, 1: Location-1, 2: Location-2, a: pre-remediation sample, b: post-remediation sample; Dust comb: combined data from settled dust samples; BM-1: building material pool from Index-1 building. Construction of clone library from the Index-2 building material pool failed.

A total of 1081 full-length fungal Internal Transcribed Spacer region of nuclear ribosomal DNA (nucITS) sequences were obtained from the eight dust samples. Fungal sequences clustered in 305 OTUs, of which 180 were singletons. The number of observed OTUs (corresponding roughly to fungal species) varied from 21 to 98 per sample, while the theoretical total OTU richness by ACE estimator varied from 67 to 298 per sample (Table 1). Rarefaction curves and ACE percentage coverage values indicated that sampling coverage was partial (Additional file 1 Fig. S1 and Table 1). Of the 305 OTUs, 33% were annotated to species, 25% to genus and 37% to class. We identified representatives of 94 genera among the OTUs that were annotated to species or genus level. Ascomycetes accounted for the majority of the total diversity in dust (52% of all OTUs, 38-88% of clones in individual libraries), the most abundant and prevalent OTUs being allied to the classes Dothideomycetes, Eurotiomycetes and Leotiomycetes. Basidiomycetes were also consistently present in the samples (44% of OTUs, 11-54% of clones), with Agaricomycetes, Exobasidiomycetes and Tremellomycetes being the most common class affiliations. The detected classes and their relative abundances across samples are presented in Figure 1. The average ratio between ascomycetous and basidiomycetous clones (N_Asc_:N_Bas_) was 3.03 for all samples, 3.47 (0.71-7.96) for reference samples, 2.15 (1.88-2.41) for samples taken from damaged buildings before renovation, and 1.84 (0.85-2.84) for samples taken from damaged buildings after renovation. The majority of fungi observed (73% of clones) shared the highest similarity with filamentous taxa. Sequences affiliated with yeast-like and lichen-forming species were also present (24% and 2% of sequences, correspondingly).

**Table 1:** Fungal diversity and concentrations in house dust samples

Sample^a^	nucITS clone library analysis^b^						Culture	qPCR^c^			Erg^d^
	
	N	**S**_**obs**_	%C	**S **_**ACE**_	*H*'	*D*	total cfu **g**^**-1**^	**S**_**qPCR**_	**total CE g**^**-1**^	ERMI value	μg/g
In1a	225	98	45	220	4.06	0.027	9.6·10^4^	12	1.4·10^7^	4.0	2.6
In1b	100	62	44	142	3.94	0.014	5.7·10^3^	6	4.4 ·10^5^	-0.7	0.4
Re1a	207	45	44	103	2.22	0.31	2.5·10^6^	9	1.3 ·10^7^	-5.2	5.5
Re1b	26	21	31	67	2.97	0.018	1.4·10^2^	9	4.0·10^5^	1.0	0.2
In2a	100	37	48	77	2.73	0.148	1.7·10^6^	17	1.2·10^7^	4.4	1.1
In2b	119	42	25	167	2.68	0.186	1.1·10^6^	22	2.6·10^6^	4.3	1.1
Re2a	167	48	52	93	2.95	0.108	1.4·10^5^	10	3.2·10^7^	-1.3	1.9
Re2b	137	75	25	298	3.88	0.030	2.7·10^5^	24	4.1·10^6^	4.6	2.6
Combined data	1081	305	45	675	4.63	0.028		33			

^a) ^Sample name abbreviations: In: index building, Re: reference building, 1: Location-1, 2: Location-2, a: pre-remediation sample, b: post-remediation sample.

^b) ^Abbreviations: N: number of clones; S_obs_: number of observed OTUs; %C: percentage coverage (ACE); S_ACE_: total no. of OTUs according to ACE richness estimator; *H*': Shannon diversity index; *D*: Simpson diversity index.

^c) ^Abbreviations: S_qPCR_: number of qPCR assays giving positive results; total CE: sum of cell equivalent counts for 69 common indoor fungi, ERMI: Environmental Relative Moldiness Index^.^

^d) ^Concentration of ergosterol.

Of the 127 unknown OTUs (OTUs not annotated to species or genus) 36 were found from several independent samples in the present material or shared a high (> 98%) sequence similarity with environmental sequences from previous studies (see Additional file 2 Table S1 for details). The most abundant individual unknown OTUs (OTU 409, 423, 446) were affiliated to class Dothideomycetes and shared low (82-88%) sequence similarities with *Colletogloeopsis blakelyi*, *Phaeotheca fissurella *and *Hortaea werneckii*. In addition to the fungal sequences, the libraries contained approximately 800 non-target sequences mostly affiliated with plant taxa, including deciduous trees (mainly *Acer platanoides *and *Betula alba*), grasses (*Poa trivialis*), cultivated plants (*Lycopersicon esculentum*, *Cucumis sativus*) and house plants (*Ficus *sp.). The number of chimeric sequences (three - 0.3%) in dust libraries was low.

Despite the high diversity and low level of dominance in clone libraries, a group of about 20 abundant genera was distinguishable, which altogether accounted for approximately 50-80% of all clones in each library (Table 2). The most dominant groups were of filamentous ascomycetes: *Penicillium *spp. (consisting largely of the *P. chrysogenum *group and *P. commune *group), *Cladosporium *spp. (*C. sphaerospermum *group, *C. cladosporioides *group and *C. herbarum *group), *Aureobasidium *and *Hormonema *(*A. pullulans*, *H. dematioides *and *Hormonema *sp.), *Phoma *(*P. herbarum *and *P. macrostoma*), *Leptosphaerulina chartarum *and *Botrytis *sp.; yeasts (*Cryptococcus *spp., *Malassezia *spp., *Saccharomyces cerevisiae *and *Candida spp*.); and rusts (*Thekopsora areolata *and *Melampsoridium betulinum*). A full list of phylotypes along with information on their annotation and frequency of detection across samples is given in Additional file 2, Table S1.

**Table 2:** The percentage frequencies of the most abundant fungal genera in the dust clone libraries.

Genus	Location 1				Location 2			
	
	In1a	In1b	Re1a	Re1b	In2a	In2b	Re2a	Re2b
**Filamentous Ascomycetes**								
*Penicillium *	0.9%	1.0%	ND	ND	49.0%	46.2%	3.0%	4.4%
*Cladosporium*	8.4%	10.0%	64.7%	ND	5.0%	8.4%	1.2%	5.8%
*Aureobasidium*	5.3%	3.0%	2.4%	7.7%	3.0%	0.8%	3.0%	15.3%
*Hormonema*	1.8%	ND	2.9%	15.4%	2.0%	0.8%	0.6%	0.7%
*Phoma*	1.3%	6.0%	1.4%	ND	ND	3.4%	1.8%	0.7%
*Leptosphaerulina*	4.4%	4.0%	2.9%	ND	2.0%	ND	ND	ND
*Botrytis*	1.8%	ND	ND	ND	4.0%	0.8%	0.6%	4.4%
*Acremonium*	ND	ND	1.0%	ND	ND	ND	ND	9.5%
*Fusarium*	1.3%	ND	ND	ND	ND	ND	7.8%	0.7%
*Phaeosphaeria*	ND	ND	ND	3.8%	ND	ND	ND	ND
*Epicoccum*	2.7%	ND	ND	ND	1.0%	ND	ND	ND
**Yeasts**								
*Cryptococcus*	4.0%	12.0%	5.3%	3.8%	6.0%	5.9%	4.8%	12.4%
*Malassezia*	3.1%	12.0%	ND	19.2%	1.0%	1.7%	5.4%	7.3%
*Saccharomyces*	ND	1.0%	ND	ND	ND	ND	43.1%	1.5%
*Candida*	1.3%	2.0%	ND	ND	ND	ND	0.6%	3.6%
*Rhodotorula*	ND	1.0%	1.0%	ND	ND	1.7%	3.6%	ND
*Mrakia*	ND	ND	ND	ND	ND	0.8%	4.8%	0.7%
*Cystofilobasidium*	0.4%	1.0%	ND	3.8%	ND	ND	ND	0.7%
**Filamentous Basidiomycetes**								
*Thekopsora*	11.1%	ND	ND	ND	2.0%	ND	ND	ND
*Rhizoctonia*	ND	ND	ND	7.7%	ND	ND	ND	ND
*Clitocybe*	ND	ND	ND	3.8%	3.0%	ND	ND	ND
*Melampsoridium*	4.0%	2.0%	ND	ND	1.0%	ND	ND	ND
*Antrodia*	ND	6.0%	ND	ND	ND	ND	ND	ND
**Other (sum of rare and unknown genera)**	48.0%	39.0%	18.4%	34.6%	21.0%	29.4%	19.8%	32.1%

The frequencies of clones affiliated with the 23 most abundant genera are shown individually. The abundant genera accounted altogether for 52-81.6% of the clones in individual libraries.

ND: not detected

#### Fungi in building material samples

Full- or near full-length nucITS sequences were obtained from 67 pure cultures and 148 clones. The clone library constructed from Index-1 building material samples contained a considerable number of ambiguous sequences, essentially chimeras, ligated double-products and putative artificial microheterogeneity, which were manually excluded from downstream analyses. The construction of the clone library from Index-2 building material DNA failed due to a low-quality amplification product. A total of 45 fungal phylotypes were identified, of which 39 were represented by cultured isolates, 11 by clones and 5 by both cultures and clones. Detailed information of the phylotypes and their isolation sources is given in Additional file 3, Table S2.

The fungi detected from building materials via cloning and sequencing of isolates were mainly filamentous species. The Index-1 building yielded solely filamentous species, most of which were xerophilic soil fungi (e.g. *Aspergillus conicus*, *Eurotium *sp., *Penicillium citreonigrum*, *P. corylophilum *and *Wallemia *sp.), whereas species favouring high water activity were identified from the Index-2 building (e.g. *Phoma *sp., *Trichoderma citrinoviride*, *T. atroviride*, and yeasts like *Cryptococcus *spp., *Sporidiobolus salmonicolor *and *Rhodotorula mucilaginosa*). Several morphologically unidentifiable (sterile) colonies were readily identified to species level by nucITS sequence analysis, including *Hormonema dematioides*, *Phoma herbarum*, *Pithomyces *(*Leptosphaerulina*) *chartarum *and *Rhinocladiella atrovirens*. All colonies provisionally identified as *Aureobasidium*-like were found to represent other taxa by nucITS-sequencing (see Additional file 3, Table S2 for details).

#### Comparison of molecular methods and culture

The fungi most abundant and prevalent by cultivation (Additional file 4, Tables S3_S4) and qPCR (Additional file 4, Tables S3_S4) methods in dust samples were largely overlapping with those observed to be abundant by clone library analysis, yet their relative abundances in individual samples did not correlate well between methods. *Cladosporium*, *Aureobasidium*, *Penicillium*, Sphaeropsidales, yeasts and unidentifiable (sterile) isolates, i.e. the dominant taxa based on clone analysis (Table 2), accounted for 89-100% of total colony forming units (CFUs) in all but one sample. A total of 13 genera were detected by cultivation, while 33 qPCR assays representing 13 genera gave a positive result from one or more samples (Additional file 4, Tables S3_S4). Of the 13 genera detected by cultivation, nine were also detected by qPCR, three were not targeted, and one (*Alternaria*) gave a negative result but was found to be represented by species (*A. citri *and *A. arborescens*) other than the one targeted by the assay (*A. alternata*).

The analytical sensitivity of qPCR was clearly superior to the clone library analysis: In 92% of cases when a qPCR-detectable phylotype occurred in a clone library, it was correctly detected by qPCR from the same sample. At the same time, only 40% of positive qPCR detections were repeated by clone library analysis (Table 3). The quantitative correlation between the methods was assessed by calculating the Spearman rank correlation coefficient for double positive detections (n = 35). The Spearman rank correlation was moderate (0.59, p < 0.01). The median concentration of species not detected by sequencing was 1.4 × 10^4 ^CE g^-1 ^and 1.7 × 10^5 ^CE g^-1 ^for species detected by sequencing. The concentrations of species detected as singletons in clone libraries varied from 1.4 × 10^3 ^CE to 5.9 × 10^5 ^CE g^-1 ^(median 5.5 × 10^4 ^CE g^-1^; Additional file 5, Fig. S2).

**Table 3:** Qualitative comparison of qPCR and clone library sequencing for detecting fungal species in dust samples

Result	No. of cases
Positive detection of a taxon in a sample by both qPCR and clone library sequencing	35
Negative result by both methods	443
Detection by qPCR only (clone library non-detect)	74
Detection by clone library sequencing only (qPCR non-detect)	4

### Comparison of fungi in moisture-damaged and reference buildings

#### Differences between fungal assemblages in moisture-damaged and reference buildings before renovation

The amount of fungal biomass, as determined by ergosterol content of dust, concentrations of culturable fungi or the summed total CE counts of common indoor molds as determined by qPCR did not show a consistent trend in relation to the presence of water damage (Table 1). In Location-1, fungal diversity was higher in the damaged building than in the reference; culturable diversity, the number of positive qPCR assays, as well as molecular diversity in the clone libraries were higher for the index building than the reference building (see Table 1 and Table 2 and Additional file 4 Tables S3_S4 and Additional file 1 Fig. S1). In Location-2, qPCR assayed diversity was somewhat higher in the damaged building, while cultivated fungi and clone library analysis indicated lower diversity for the index building than the reference (Table 1 Additional file 4 Tables S3_S4). Dust culture plates and clone libraries from the Index-2 building yielded notably high counts of *Penicillium *(*Penicillium chrysogenum *group colonies and two OTUs affiliated to *P. chrysogenum *and *P. commune *groups, correspondingly), which may have masked the presence of other fungi (Additional file 4 Tables S3_S4).

**Figure 2:**
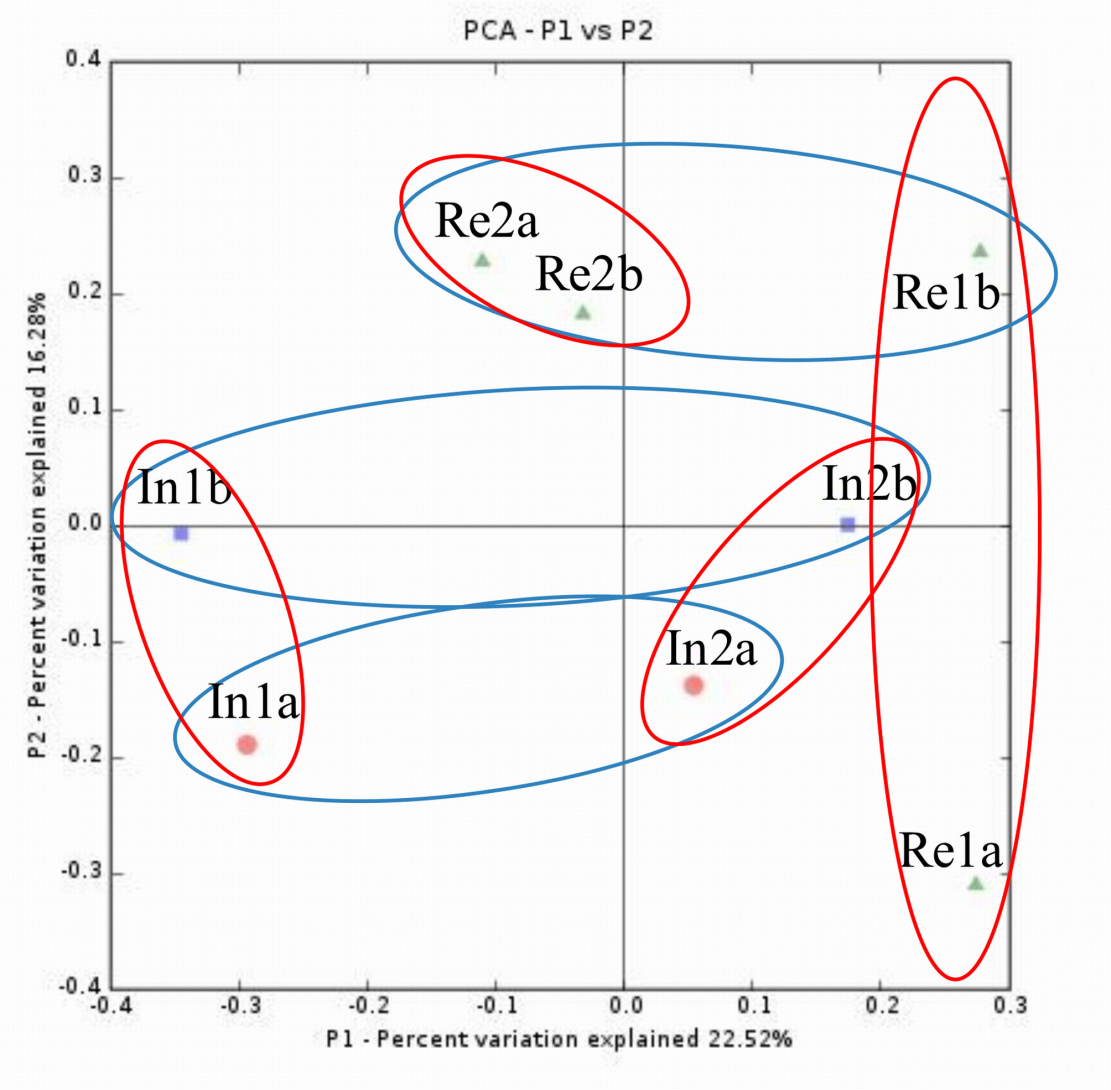
**UniFrac PCoA of dust sample nucITS library clone frequencies**. The first and second principal coordinates (P1 and P2) are shown. The first axis correlates with building (P1, red circles, 23% of variation). Apart from reference sample Re1a, the second axis correlates with building conditions (P2, blue circles, 16% of variation). The circles were drawn manually.

β-diversity indices, the UniFrac program distance measurement and a PCoA analysis were used to determine the pairwise similarities of clone library compositions of index and reference buildings. The proportions of shared OTUs (i.e. species in common) were, in general, low between buildings; the QS values varied between 0.09 and 0.21. The two index buildings shared the highest proportion of common OTUs, and the two reference buildings the lowest. According to the UniFrac significance test, all sample pairs, except for the two index buildings, differed from each other significantly at the time of pre-remediation sampling (Additional file 6 Table S5). The first coordinate (P1) found in the UniFrac PCoA analysis separated samples by building, explaining 23% of the variation. The second coordinate (P2, 16% of the variation) separated the pre-remediation samples from index buildings from post-remediation index samples and most of the reference samples, suggesting that the presence of moisture damage may have contributed to the altered phylogenetic composition of the fungal communities in dust (Figure 2).

**Figure 3:**
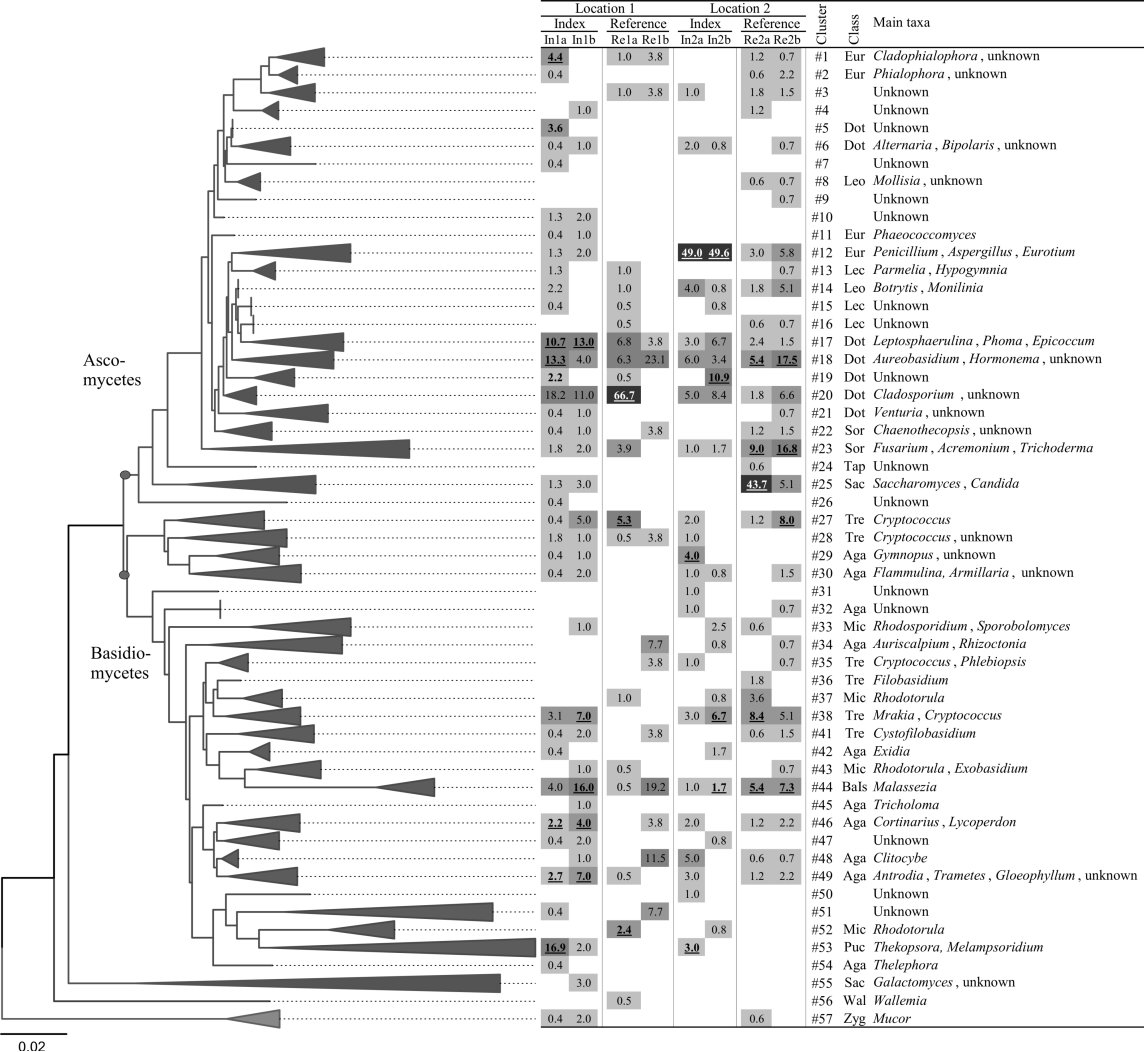
**Phylogenetic representation of indoor dust fungal communities inferred from nucITS clone library data**. Percentage frequency representation of clusters in individual dust samples are given as a heat map table, also showing cluster numbers (#), class and main genera included. A statistically significantly increased occurrence of a cluster in a sample is shown underlined (UniFrac analysis). Class abbreviations: Eur: Eurotiomycetes; Dot: Dothideomycetes; Leo: Leotiomycetes; Lec: Lecanoromycetes; Sor: Sordariomycetes; Tap: Taphrinomycetes; Sac: Saccharomycetes; Tre: Tremellomycetes; Mic: Microbotryomycetes; Aga: Agaricomycetes; BaIs: Basidiomycetes incertae sedis; Puc: Pucciniomycetes; Wal: Wallemiomycetes; Zyg: Zygomycetes incertae sedis. For detailed cluster contents and OTU annotations, see Additional file 2 Table S1.

**Figure 4:**
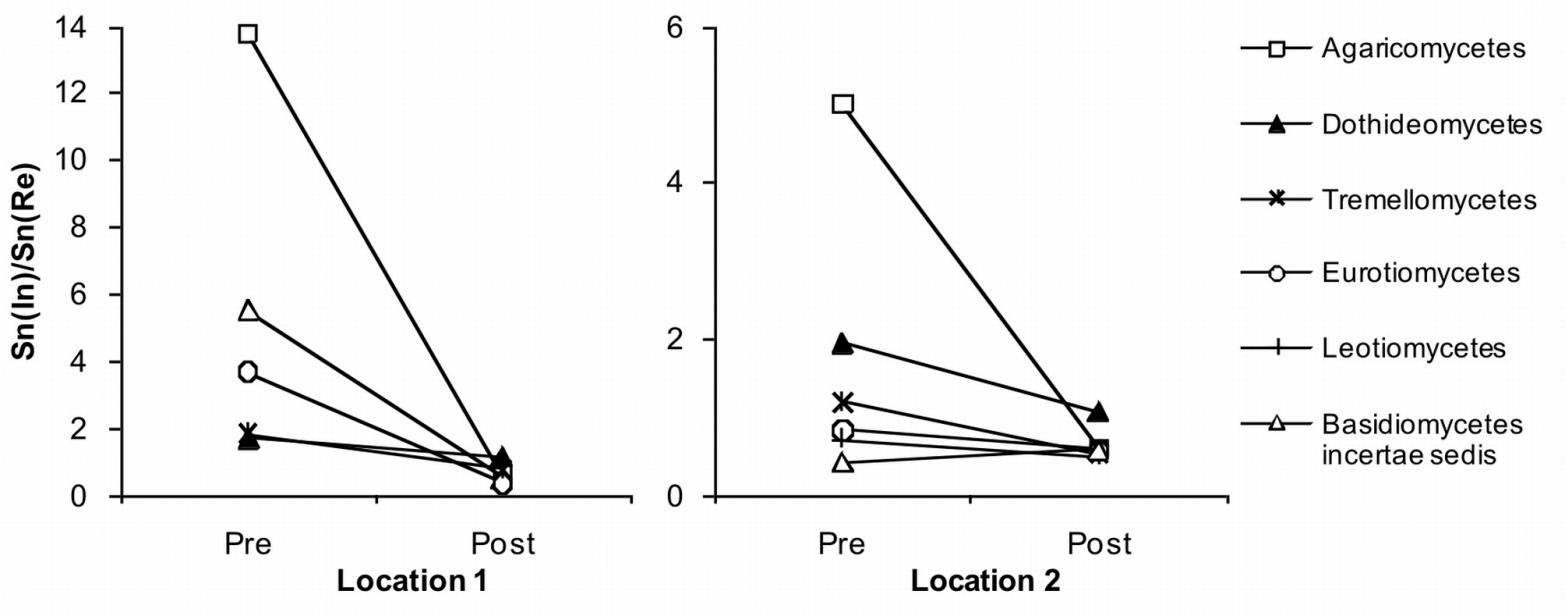
**Pair-wise comparison of fungal species richness in water-damaged and reference buildings pre- to post-remediation**. Phylotype diversities (S_n_) were calculated from clone library data separately for each sample and for each fungal class. The diversity ratio between the index and reference buildings (S_n(In)_:S_n(Re)_) was calculated for each building pair pre- and post-remediation. The results for the two locations are shown separately. The species richness of Agaricomycetes, Eurotiomycetes and Dothideomycetes was higher in the index buildings in relation to reference buildings' pre-remediation, but decreased post-remediation.

The UniFrac program was subsequently used to conduct a tree-based analysis to determine which fungal clusters occurred in individual samples at a significantly higher frequency than expected (compared to random OTU distribution). The results of this analysis are presented in Figure 3; the detailed OTU composition of the clusters shown in the figure is given in Additional file 2 Table S1. Ten phylogenetic clusters (clusters # 1, 5, 12,17-19, 29, 46, 49 and 53) occurred in one or both index buildings at a higher than expected frequency. The Index-2 building was heavily dominated by *P. chrysogenum- *and *P. commune*-related OTUs (cluster 12). In contrast, several clusters (# 1, 5, 17-19) of diverse ascomycete OTUs were characteristic of the Index-1 building. These clusters were affiliated with the classes Dothideomycetes and Eurotiomycetes, and included known colonizers of indoor materials (e.g. *Aureobasidium pullulans*, *Cladophialophora minutissima*, *Exophiala xenobiotica*, *Epicoccum nigrum*, *Leptosphaerulina chartarum*) as well as a variety of related, unknown OTUs. Similarly, the basidiomycete clusters characteristic of index buildings (# 29, 46, 49) included potentially building-associated species, e.g. *Serpula lacrymans*, *Gloeophyllum sepiarium *and *Trametes versicolor*, yet these phylotypes occurred at a low frequency. Other lineages were associated with the reference buildings. These contained *Cladosporium- *and *Aureobasidium*-related Dothideomycetes (# 18, 20) as well as Sordariomycetes (# 23, mainly *Fusarium oxysporum*) and various yeasts including *Cryptococcus *spp., *Mrakia *spp. and *Rhodotorula *spp. *S. cerevisiae*, (# 27, 38, 52 and 25, correspondingly). The within-class phylotype richness ratio was elevated (S_n(In)_/S_n(Re) _= 1.7-13.8) among classes Agaricomycetes, Dothideomycetes and Tremellomycetes in both index buildings in relation to their references (Figure 4).

Table 1 shows the ERMI values derived from the qPCR data. These were higher for the index buildings (4.0 and 4.4) and lower for the reference buildings (-5.2 and -1.3). The following group 1 ERMI assays were responsible for elevated values in the index buildings: Wsebi, PvarB, Tviri (Index-1) and PenGrp2 (Index-2).

#### Occurrence of material-associated fungi in dust

A total of 45 fungal phylotypes were detected from the building material samples collected from the two index buildings. An *in silico *analysis showed that 13 of the phylotypes (29%) had a matching sequence with the qPCR targets (see Additional file 7 Table S6 for targeted species). Eight of the 45 phylotypes were detected in the dust samples in corresponding buildings using clone library analysis or qPCR. These were *C. cladosporioides*, *C. herbarum*, *Eurotium *sp., *P. chrysogenum*, *P. herbarum*, *P. chartarum*, *T. atroviride *and *W. sebi*. Most of these were ubiquitous in both the index and reference buildings' dust samples. The summed qPCR cell counts for these fungi were similar in the index and reference building pairs; together, the species accounted for 3.8 × 10^5^/8.0 × 10^5^CE g^-1 ^and 6.4 × 10^5^/6.7 × 10^5^CE g^-1 ^in the index/reference buildings in Location-1 and Location-2, correspondingly. Three individual taxa, *L. chartarum*, *T. atroviride *and *W. sebi *occurred exclusively, or in substantially higher numbers, in an index building than the corresponding reference building (Additional file 2 Table S1). *Penicillium chrysogenum *was abundant only in the index building according to clone library analysis, but qPCR reported equally high numbers of this species in both the reference and the index buildings.

### Changes in fungal assemblages in dust after renovation

We monitored the qualitative and quantitative pre- to post-remediation changes in fungal community structures in dust samples collected from the index buildings in relation to the changes occurring in corresponding reference buildings. The results are reported separately by location.

#### Location 1

In both the index and reference building at Location 1, the levels of fungal biomass (as indicated by ergosterol content in the dust), culturable fungi and concentrations of common indoor fungi as enumerated by qPCR were lower post- than pre-remediation (Table 1). Fungal diversity as inferred from the number of positive qPCR assays, as well as from the level of molecular diversity (Table 1 and Additional file 1 Fig. S1), decreased after remediation in the index building. In the reference building, the number of positive qPCR assays was similar pre- and post-remediation, while the change in molecular diversity was not clear due to the small clone library size. The phylotype richness ratio of the buildings (S_n(In)_/S_n(Re)_) was lower for all fungal classes post-remediation (Figure 4). The ERMI value was lower post-remediation in the index building (change from 4.0 to -0.7) but higher (from -5.2 to 1.0) in the reference building (Table 1).

Most of the fungal lineages identified by the UniFrac lineage analysis to be specific for the Index-1 building pre-remediation disappeared (clusters # 1, 5 and 19), or had decreased in abundance (# 17, 18 and 53) following remediation. Concerning the occurrence of material-associated fungi in dust, *T. atroviride *and *W. sebi *were not found in the post-remediation sample by qPCR or clone library sequencing. The proportion of the *L. chartarum *phylotype instead remained unchanged in clone library pre- to post-remediation. The PCoA analysis separated the pre- and post-remediation samples taken from the Index-1 building, and suggested a small shift in community composition towards the reference buildings' composition along the second coordinate (Figure 2).

#### Location 2

The pre- to post-remediation changes in the levels of fungal biomass, culturable fungi and summed concentrations of qPCR-assayed indoor fungi in Location-2 were similar in the index and reference building (Table 1). Fungal diversity was higher post- than pre-remediation in the reference building but not in the index building. Diversification in the reference building was seen in the elevated numbers of culturable genera, positive qPCR assays (Additional file 4 Tables S3_S4) and ERMI values, as well as in clone library-derived diversity indices and rarefaction analysis (Table 1 and Additional file 1 Fig. S1). UniFrac PCoA analysis and pairwise Sørensen similarity values indicated that, despite the diversity increase, both the OTU-based and phylogenetic community structure remained very similar pre- to post-remediation in the reference building. The species richness of prevalent fungal classes was lower in the Index-2 building in relation to the reference; the within-class phylotype richness ratios (S_n(In)_/S_n(Re)_) for Agaricomycetes, Dothideomycetes and Tremellomycetes, which were elevated before remediation, were close to or below one after remediation (Figure 4). Somewhat contrastingly, several fungi initially isolated from the building materials but absent during initial dust sampling were observed following remediation (e.g. *Hormonema dematioides*, *Phoma *sp., *Rhodotorula mucilaginosa*, *Cryptococcus adeliensis*). The abundance of the dominant clade (# 12, *P. chrysogenum *group) in the Index-2 building did not change following remediation (Figure 3, Additional file 2 Table S1).

## Discussion

To our knowledge, this is the first time that the effect of moisture and moisture damage remediation on indoor fungal assemblages has been studied using a whole community approach and source tracking. It is also the first study to compare fungal community composition using a large selection of species-specific qPCR assays and clone library sequencing in combination with culture. We found increased fungal diversity in one of the studied buildings with moisture damage, while in the second damaged building, high numbers of *Penicillium *were present. In neither building did we find a concomitant increase in culturable fungal concentrations or fungal biomass in surface dust. A majority of the fungal species isolated from contaminated building materials was not prevalent in the pre-remediation dust samples collected from those buildings. Methodological comparison indicated that cultivation in combination with a large qPCR panel, failed to detect a majority of the fungi in indoor samples; however, the most abundant species appeared to be detected by all methods. Clone library sequencing, to the extent used here, was found to be less sensitive than qPCR for detecting individual species.

### Fungal diversity in dust samples

Cloning and sequencing studies revealed an average of 54 observed and 146 estimated species-level phylotypes (OTUs) per sample. This level of diversity is similar to that observed previously using molecular methods in floor dust and indoor air filter samples [21-23] and higher than that detected in outdoor air filter samples [27,28].

The dominant genera we observed in dust and material samples were in agreement with previous studies using cultivation [29-32]; *Aureobasidium*, *Cladosporium *and *Penicillium *were the most prevalent genera in dust according to molecular and culture-independent methods. These and other common indoor mold genera, including *Aspergillus*, *Botrytis*, *Epicoccum*, *Eurotium*, *Fusarium*, *Mucor*, *Rhizopus*, *Trichoderma*, *Ulocladium*, *Wallemia *and *Phoma*/Sphaeropsidales-group fungi accounted for 95-96% of total CFUs and qPCR CE counts and approximately 40% of clones in nucITS libraries. The remaining 60% of nucITS clones, however, accounted for almost 90% of the total diversity in the sequence material, showing that a vast diversity of indoor fungi remain uncharacterized by cultivation or targeted molecular methods. While the proportion of individual sequence types representing the uncultivable diversity was low in the material, it must be remembered that the clone library sequencing method does not accurately reflect the original proportions of species in the community and both under- and over-estimating bias may occur [33]. Our results from individual qPCR assays indeed showed that the species occurring as singletons in nucITS libraries were in many cases abundant taxa, commonly between 10^4^-10^5 ^CE g^-1 ^of dust. According to previous data from Finland and the US, the median qPCR assayed concentrations of many common indoor fungi, e.g. *Aspergillus *spp., *Epicoccum nigrum*, the *Eurotium amstelodami *group, *Penicillium *spp. and *Trichoderma viride *are between 10^4 ^and 10^5 ^CE g^-1 ^of floor dust [18,34]. No such data are available for settled dust collected from elevated surfaces, but the fungal concentrations in the latter sample type can be expected to be similar or lower than those in floor dusts [22,35].

Based on the number of described fungal species [36] and estimates on total global fungal biodiversity [37] nearly 90% of fungal biodiversity may as yet be unidentified. A large proportion of unidentifiable phylotypes was observed in our sequence material also. In total, 42% of OTUs could only be identified to the class or phylum level, or remained of unknown affiliation. This is comparable to previous studies reporting 16-62% unidentified fungal OTUs from diverse environments [27,38,39]. While artefactual sequence motifs, resulting from polymerase errors and chimera or heteroduplex formation are known to occur in clone libraries [33,40], we are confident that the number of such sequences was low in our material because of our prior efforts to optimize PCR conditions [23]. 36 unknown OTUs occurred in several samples in the present material or matched with unknown environmental phylotypes from previous studies. At least, these 36 sequences most probably represent natural phylotypes, because the formation of a unique artefactual PCR product from diverse template pools independently more than once would be highly unlikely. Interestingly, about one fifth of the unknown OTUs were found in indoor samples collected from the same geographic region in our previous study [23]. A novel phylotype related to skin-associated lipophilic yeast genus *Malassezia *(with 79% sequence similarity to *M. sympodiales*) detected previously [23] was prevalent in the present material. Moreover, several clusters of unknown filamentous ascomycetes were found. Some were affiliated with common indoor taxa capable of growing on indoor materials. This suggests that it is possible that building materials may also harbour yet to be identified fungal species.

Besides unknown ascomycetes, Basidiomycetes and yeasts accounted for a substantial part of the unculturable majority of nucITS sequence diversity. These are common in culture-based studies as well, but cannot be routinely identified by morphology [41-43]. While the significance of building-associated filamentous basidiomycetes relates to their wood-decomposing ability rather than human health effects, these fungi may have an indicator value in building investigations, and thus may be important targets for designing molecular diagnostic tools [44]. In the present study, certain building-associated basidiomycetes including *Serpula lacrymans *(the causative agent of timber dry rot), *Antrodia sitchensis*, *Trametes versicolor *and *Gloeophyllum sepiarium *[45,46], were found, mostly from the water-damaged, wood-framed Index-1 building. These species may have had an intramural source also in the present study. However, this connection could not be verified by examination of the building materials.

Several opportunistically pathogenic taxa [47] were also identified, including *Candida zeylanoides*, *Cryptococcus albidus*, *Exophiala xenobiotica*, *Mucor *spp. and *Trichosporon mucoides*.

In addition to a wide diversity of fungi, we also found DNA signatures of an impressively diverse array of plants including cultivated crops (fruits, vegetable crops and tobacco), deciduous trees, grasses, mosses and weeds. The amplification of plant DNA was likely due to a lack of specificity in our forward PCR primer [23]. Despite the fact that the inclusion of plant targets was not our intent, their recovery further confirms the biological complexity of dust, and indicates that DNA-based methods may be useful for the detection of dust-borne plant particles. Like fungal particles, those originating from plants may also have allergenic potential, and obviously persist in indoor dust, long past the respective pollen season.

The representativeness of different dust sample types has been discussed in the context of airborne exposure analysis; for example, the presence of heavy, non-resuspending particulate material in floor dusts, as well as potential microbial proliferation in dusts collected from locations with elevated relative humidity have been suspected to bias dustborne measurements [48-50]. A comparison of our above-floor surface samples with floor dust samples collected earlier during the cold season from the same geographic region [23] indicated differences in fungal community composition. Especially, lower frequencies of basidiomycetous yeasts (mainly *Malassezia *and *Cryptococcus*) and rusts were found in dusts collected from elevated surfaces. This difference was also reflected in the differential ratios of Ascomycetes and Basidiomycetes (N_Asc_:N_Bas_) between the two sample types; the average N_Asc_:N_Bas _ratio was 3.03 for the elevated surface dust, but lower (0.95) for floor dust. The differences may relate to the aerodynamic properties of different fungal particles; while the spores of the mentioned genera are not distinguishingly large, they are commonly carried along with larger particles (i.e. *Malassezia *cells on human skin scales and *Cryptococcus *cells on plant debris), which makes them more prone to deposit on floor surfaces. In contrast, many ascomycetous particles are small, air-dispersed microconidia that stay airborne for long periods, resuspend efficiently and deposit on elevated surfaces. This finding fits into the hypothesis of differential size distribution of particles in the two sample types, and suggests that the small particle fraction may be better represented in elevated surface samples.

Local fungal amplification may have a significant biasing effect on fungal measurements of the dust samples [48,49]. Our findings suggest that microbial proliferation in settled dust itself had not been extensive in the studied conditions. This was supported by the high molecular diversity coupled with the low dominance of individual OTUs, a strong contribution of species unable to proliferate in indoor habitats and a generally low proportion of *Aspergillus*, *Eurotium *and *Penicillium *(genera known to proliferate efficiently in dust in elevated humidity; [47]). This dust type seems to act as a sink for fungal propagules arising from various sources, as previously suggested by Scott et al. [49]. These observations may yet hold for temperate regions only; differential observations were made by Amend *et al*. [21] from dust samples collected from the tropics with higher relative humidity; there *Aspergillus*, *Eurotium *and *Wallemia *were prevalent, and the overall molecular diversity was lower. The observations by Amend *et al*. [21] from temperate regions were similar to ours.

### Fungal diversity in building material samples

The spectrum of fungi in building material samples was very different from that observed in dust: Practically all phylotypes were affiliated with filamentous ascomycetes and only a few with basidiomycetes, all of which were yeast-like species. The number of phylotypes observed in material samples was low compared to dust samples. This may have been partly caused by technical problems in the clone library construction; it may also reflect the profound differences of these substrata. While dust acts as a repository of particles, wet building materials support a limited set of taxa, probably as a function of restrictive nutritional characteristics of the substrata and interference competition. The phylogenetic spectrum of fungi observed by sequencing was similar to that observed by cultivation; both methods showed a predominance of taxa affiliated with Dothideomycetes, Eurotiomycetes and Leotiomycetes.

The analyzed building material samples were collected from two moisture-damaged buildings of different construction types. The community composition differed in the two buildings: The Index-1 building was dominated by filamentous xerophilic soil fungi, whereas plant and wood-associated species favouring higher water activity, including yeasts, predominated in samples from the Index-2 building. While others have reported associations between fungal genera and building material types [41], such separation was not obvious here. Instead, we hypothesize that the predominance of different fungal ecotypes was linked to the sampled building locations: Soil-associated xerophiles tended to dominate the water-damaged ground-level and below-grade sites sampled from the Index-1 building, while phylloplane fungi dominated in roof constructions sampled from the Index-2 building. However, these observations were made from a very limited number of samples, and thus need further testing with larger sample numbers.

Nearly all clones and isolates from building materials could be identified to species level by their nucITS sequences. Most of the fungi detected had been isolated from building materials before [41,51,52]. In addition, we identified several species that have not previously been reported as contaminants of building materials (e.g. *Penicillium canescens*, *Thielavia hyalocarpa*, *Cryptococcus adeliensis*). Moreover, clones and isolates without close sequence relatives in DNA databases were also found. This confirms that the present, largely cultivation-based view of building-associated fungal diversity is incomplete and should be studied in detail using cultivation-independent methods. Advanced isolation techniques using minimal selectivity [53], as well as novel massively parallel sequencing applications, may offer feasible alternatives to further elucidate this unexplored biodiversity from large numbers of samples.

### Effect of moisture damage and remediation on fungal assemblages in dust

We found higher molecular diversity and ERMI scores in dusts collected from damaged buildings than their matched references. In contrast, elevated total concentrations of fungal biomass, total cell counts of common indoor molds or culturable fungi were not seen. Visible water damage and mold growth on surfaces is often associated with elevated concentrations of fungi in dust [25], but low levels in dust are not uncommon when the growth is located inside the building envelope [26], as was the case in the present study.

The increased diversities in index buildings were associated with fungal classes that include building inhabiting decomposers (Agaricomycetes) and saprotrophic molds (Dothideomycetes and Eurotiomycetes); elevated ERMI scores suggested an increase in water-associated fungi in index buildings. Despite this, few of the fungi detected from the water-damaged building materials were actually found in the corresponding dust samples, even using the combination of qPCR (a sensitive technique) and clone library sequencing (a non-selective technique). This may indicate that the transfer of DNA containing cell material from the site of growth to the room space was not remarkable compared to other fungal sources. On the other hand, the low number of shared taxa between materials and dust may have been a consequence of undersampling of materials from contaminated building sites and/or the failure to construct clone libraries from individual material samples. We used 69 different qPCR assays to study the fungi in dust, but this selection covered less than one third of the 45 phylotypes found in materials. Thus, it is possible that a larger proportion of the observed fungi in dust was attributable to building material sources than could be verified here.

Remedial and cleaning efforts were associated with a decrease in the diversity of dustborne fungi in one of the buildings. This, as well as the disappearance of certain material-associated species, supports the assumption that remediation was effective in the removal of the fungal burden contributed by indoor mold growth sources. In the second location, clear indications of an intervention effect on the diversity were not seen. Due to a delay in remediation schedules the interval between completion of the remediation and post-remediation sampling was short, which may explain the increase in the abundance of material-associated fungi in post-remediation dust; despite efforts to prevent the spread of contamination, fungal particles aerosolized during remediation may have spread, not being sufficiently removed by post-remedial cleaning. In addition, there was an unexpected diversification in the reference building's microbial profile, which undermined the case-control comparison. The diversification may have been caused by an increase in the transfer of fungal material from outdoors. This hypothesis is supported by the appearance of many probably outdoor-related phylotypes in the clone libraries. Yet the diversification included many species that may proliferate indoors, and thus the occurrence of water damage in the reference building cannot be ruled out. In Location-2, the considerable distance between the index and reference buildings also challenged the comparison. These findings highlight the strong variation in indoor mycobiota within and between buildings, the uniqueness of individual buildings' microbial profiles and the complexity of potential sources. For these reasons, the choice and matching of reference building for each study building is crucial. In general, our findings are only suggestive due to the deep normal variation between buildings and the small building number, and should be further examined with larger data sets.

### Comparison of methods

Of all methods tested, clone library analysis provided the most thorough inventory of fungal diversity in settled dust. Nevertheless, a comparison of the sequencing results with qPCR results (a technique with higher analytical sensitivity) showed that many species present in the samples were not represented by the libraries. The species only detected by qPCR tended to be those of lower qPCR cell counts, whereas highly abundant species were much better represented in the clone libraries.

Taking into account the semiquantitative nature of clone library results and the presently deficient species-level information of potential building-associated fungi, the usefulness of clone library sequencing for assessment of building sources remains uncertain. This uncertainty also arises from the universal nature of the technique, i.e. its sensitivity in detecting background diversity acting as a dampening factor on the ability to detect shifts in indicator species. Novel highly parallel sequencing techniques like 454 pyrosequencing overcome the limitations of sensitivity, but the quantitative representativeness remains a problem [21]. In the present study, despite its selectivity, plate cultivation was partly successful in reflecting increased fungal diversity and/or detecting major indicator fungi arising from building material sources in settled dust samples. This was not, however, consistent across all samples, as the masking effect of certain species occurring in very high concentrations was considerable.

ERMI is an index derived from a set of qPCR assays used to describe the indoor fungal burden [20]. Here, the ERMI values were below 5, i.e. relatively low compared to US homes. Vesper et al. reported ERMI values greater than 5 for the highest quartile of randomly selected US homes, whereas over 75% of homes with asthmatic children were above this value [54]. However, no similar data are available in Finland. In the present study, the ERMI index was observed to reflect the overall level of diversity. In our sample material, the group 1 members *A. pullulans *and *Eurotium *spp. occurred in significant concentrations in all studied dust samples and in similar concentrations in the index and reference buildings. This suggests that the placement of these species in the indicator group may not be appropriate.

## Conclusions

The present study is the first to assess the effect of water damage and its remediation on indoor mycobiota using universal culture-independent community characterization methods, and also the first study to compare nucITS sequencing results with an extensive panel of mold specific qPCR assays. Observations were made from a small number of buildings, and thus the findings are descriptive and need to be studied further with larger data sets. In the studied buildings, we found indications of elevated fungal diversity, as well as the presence of fungi attributable to building growth to be associated with water damage. The community variation between buildings was significant, and calls for the analysis of larger data sets in order to understand the dynamics of microbial communities between building structures, surfaces and dust. Our results demonstrate that culture-based methods used to characterize indoor mycobiota provide an underestimate of the total diversity, and that many unknown or unsequenced fungal species are present in dust. Despite this, the majority of abundant phylotypes in nucITS clone libraries were affiliated with previously recognized indoor taxa, indicating that culture-dependent and independent methods agree on the dominant indoor taxa. Clone library sequencing was seen as an effective means to characterize indoor communities, and proves extremely useful when attempting to answer research questions on 'real' fungal diversity in a given environment. However, this approach is not ideal if the specific detection of fungal taxa that are indicators of microbial indoor growth is the aim, as the level of background diversity and its variation are considerable, and this approach has limitations in sensitivity and quantitative presentation of results. qPCR was found to be more sensitive than clone library sequencing in detecting specific fungi in dust. We found unknown and atypical fungi on moisture-damaged building materials, which calls for more detailed investigation of the mycobiota capable of growing on building materials.

## Methods

### Buildings

The study material consisted of two pairs of office buildings (n = 4) in two locations (*Location 1 *and *Location 2*). Of each pair, one building (the *Index-1 *and *Index-2 *buildings) had a history of moisture and mold damage coupled with health complaints from the building occupants; the second building (the *Reference-1 *and *Reference-2 *buildings) lacked a similar history. Otherwise the buildings were matched for age, construction type, usage, condition and ventilation type. The buildings of Location 1 (*Index-1 *and *Reference-1*) were wooden frame structures located in the same building complex outfitted with mechanical exhaust ventilation systems. The main sources of water in the index building had been roof leakages. The buildings of Location 2 consisted of a slab-on-grade foundation with one- or two-storey concrete formwork, and were outfitted with balanced mechanical ventilation systems. The index- and reference buildings were located approx. 100 km apart from each other. The Index-2 building was water-damaged by roof leakage and capillary migration of ground water through the basement floor slab. In the course of the study, the damaged buildings underwent a thorough remediation during which damaged components of the building, including interior finishes, insulation and parts of the framing were replaced. The sources of moisture were identified and eliminated. No intervention or extra cleaning was performed in the reference buildings. Previous work describes the mycobiota of outdoor air outside the studied buildings, where the concentrations of 22 fungal species or groups were assessed using qPCR in parallel with the measurements described in the present study [55].

### Dust and material sampling

Dust samples (n = 8) were collected twice from each of the four buildings, during consecutive winters. During the intervening summer and autumn period the index buildings were remediated and a post-remediation cleaning of the interior surfaces was performed. The interval between remediation and follow-up sampling was approximately six months in Location 1 and three months in Location 2. Reference buildings were sampled at corresponding times. Settled dust was collected and processed as described in detail previously [23]. Briefly, a long term composite sample of accumulated fine dust was obtained by vacuuming from above floor level surfaces (including the top of shelves, tables and other surfaces) twice a week for 2-6 weeks into nylon dust sampling socks. The collected dust was sieved using 1 mm mesh, homogenized and aliquoted for analyses. During remediation, moisture-damaged building material samples were collected from the two index buildings. Samples were weighed, homogenized, and microbial cells were eluted into sample buffer by sonication as described previously [41]. The material samples from Index-1 building (n = 7), included timber, wood board and mineral wool from ground floor constructions and walls, while samples from Index-2 building (n = 9) included wood and wood fibre board, concrete, mineral wool and filler from floor and roof constructions. A summary of the samples analysed and methods used to compare fungal assemblages is given in Additional file 8 Table S7.

### Determination of culturable fungi and ergosterol analysis

Culturable fungi from dust and material samples were enumerated by dilution plate culture on 2% malt extract agar (MEA) and dichloran-glycerol (DG18) agar followed by microscopic examination, as described previously [23,41]. The identification of representative isolates from materials was confirmed by sequencing the full-length nucITS region as described previously [23]. For ergosterol analysis, two replicate samples of 5 mg of dust were assayed by gas chromatography-mass spectrometry according to the method of Sebastian and Larsson [56] with minor modifications [23], and the arithmetic mean of the two replicates was calculated.

### Molecular methods

The molecular methods to describe fungal community composition, including DNA extraction from dust, optimized universal PCR amplification of full-length nucITS, and construction and sequencing of clone libraries have been described in detail previously [23]. All DNA extractions were done in duplicate. Negative PCR controls were always used. For qPCR, an external amplification control (*Geotrichum candidum *conidia) was spiked into dust samples prior to DNA extraction. For clone library construction, ten parallel PCR reactions were set up for each sample and the resulting PCR products were pooled prior to cloning. For the analysis of building materials, amplification products from individual material samples from each building were pooled prior to cloning to provide one composite product for each building. Due to very low initial PCR product yields, these composite samples from materials were re-amplified by similar PCR to yield sufficient DNA material for cloning.

The concentrations of 69 fungal species or groups of species were determined by qPCR, including assays required for the calculation of the Environmental Relative Moldiness Index (ERMI; [20]). The details of the DNA extraction for qPCR, assay protocol and controls have been described previously [23,57]. A full list of assays performed along with detected taxa is given in Additional file 7 Table S6, while the primer and probe sequences used in the assays are available online at http://www.epa.gov/nerlcwww/moldtech.htm. ERMI was calculated according to Vesper et al. [20], essentially, by subtracting the sum of logs of concentrations (CE mg^-1 ^of dust) of a set of common, non-indicator fungi from that of moisture-indicator taxa. Representative DNA sequences of recovered fungi were submitted to the EMBL Nucleotide Sequence Database [58] and assigned accession numbers FR718449-718487 and FR682142-682466 for cultivated strains and clone library phylotypes, respectively.

### Phylogenetic and statistical data analyses

Sequence data were treated as described before [23]. Phylogenetic and statistical analyses were performed using bioinformatics software freely available for academic users. Program sources are listed at the end of the corresponding reference. Distance matrixes were constructed for each sample and for the combined data from the alignments by using the DNADIST program [59]. The program package Mothur [60] was used to cluster sequences with the average neighbor method into operational taxonomic units (OTUs) with 99% similarity. Potentially chimeric sequences were identified using the program Bellerophon [61] and investigated manually. FigTree [62] was used to visualize and edit phylogenetic trees. Full-length nucITS sequences were assigned to species- or genus level based on similarity values to closest matching reference sequences in International Nucleotide Sequence Database (INSD) according to the scheme described by Ciardo et al. [63]. For OTUs having ≥ 98% similarity with an INSD reference, the annotation was refined manually when applicable. Unknown OTUs (i.e., OTUs not assigned to species or genus) were provisionally assigned to class by BLAST result and rDNA gene tree clustering. OTU richness and diversity estimates were calculated using Mothur program; rarefaction curves of the number of observed OTUs and end values from the non-parametric ACE richness estimator were used to describe theoretical OTU richness in samples. Shannon (H') and Simpson (D) indices were computed to describe OTU diversity [60]. To assess species richness within individual fungal classes, OTU richness normalized within-class (S_n_) was calculated for each class and sample by dividing the number of OTUs affiliated to certain class by the total number of clones in the library. Subsequently, the ratio of the values between index- and reference building samples (S_n(In)_/S_n(Re)_) was determined. Classic incidence-based Sørensen (QS), and Chao's abundance-based Sørensen indices for β-diversity were calculated using the EstimateS program [64] for pair-wise comparison of the OTU composition of samples. Due to variability in library size, a random selection of 100 sequences was re-sampled using R statistical environment [65] from each library apart from library Re1b from which only 26 sequences were obtained and used.

The UniFrac program was used to compare the phylogenetic content of the clone libraries [66]. UniFrac estimates microbial community similarity by pair-wise measurement of the phylogenetic distance separating the taxa unique to each sample. For this, a second sequence alignment was constructed that excluded ambiguously aligning columns in ITS-1 and ITS-2 regions, and a neighbor joining tree was created from this data set. The length of the alignment was 214 characters and the tree contained 202 unique branches. The tree was used to perform the UniFrac distance analysis, the UniFrac significance test and the Principal Coordinates Analysis (PCoA, unweighed). The UniFrac Lineage Specific Analysis option was then used to identify the fungal clades that significantly contributed to the differences in community composition between samples. The quantitative correlation between sequencing (clone library frequency) and qPCR (CE g^-1 ^of dust) results was studied by calculating Spearman correlation coefficient for pairs of positive detections. Clone library percentage frequencies were first multiplied by the sample's fungal biomass value (ergosterol concentration) to better reflect the fungal levels in the samples (F_c _= F*c[erg]). The correlation was calculated from log-transformed (X' = log10(X+1)) data in R statistical environment [65]. P-values were subsequently computed from a permutation test with 10000 random replicates.

## List of abbreviations

CE: Cell Equivalent: the unit used to express qPCR results; 1 CE usually corresponds to 1 fungal spore; ERMI: The Environmental Relative Moldiness Index: a qPCR-based technique for describing the relative abundances of moisture indicator and non-indicator species in indoor dust samples; nucITS: Internal Transcribed Spacer region of nuclear ribosomal DNA: a molecular clock like gene region that contains species specific regions; used here as a "molecular species barcode" to detect and identify species based on their DNA; OTU: Operational Taxonomic Unit: a cluster of homologous DNA sequences that share certain level of sequence similarity; used here to cluster fungal community nucITS sequences into species-level groups; PCoA: Principal Coordinates Analysis: a multivariate statistical method for finding and displaying major axes of variation among samples; used here to describe the phylogenetic similarity/distance between fungal communities in dust samples; qPCR: Quantitative Polymerase Chain Reaction: a method for gene quantification; used here to quantify predetermined sets of microbial species or higher groups based on the abundance of their species specific gene regions (mainly nucITS) in dust samples.

## Authors' contributions

MP did the cloning, sequencing and data-analyses and drafted the manuscript, TM performed the qPCR assays and edited the manuscript, AH did the ergosterol analyses and edited the manuscript, AN designed the study and edited the manuscript, LP participated in study designing and supervised the sequencing, PA edited the manuscript, UL did the culture analyses and edited the manuscript, HR collected the samples, performed the qPCR assays and edited the manuscript. All authors participated in the study design and read and approved the final manuscript.

## Supplementary Material

Additional file 1 **Fig. S1: Rarefaction curves for the analysed nucITS clone libraries**. Click here for file

Additional file 2 **Table S1: Phylogenetic description, nearest database relative and frequency of detection of fungal molecular OTUs and isolated strains recovered from dust and water damaged building material**. Click here for file

Additional file 3 **Table S2: List of fungal phylotypes obtained from building materials by cultivation and clone library analysis**. Click here for file

Additional file 4 **Tables S3 and S4: Concentrations and diversity of fungi determined by culture (S3) and quantitative PCR (S4) in dust**. Click here for file

Additional file 5 **Fig. S2: Comparison of clone library frequencies and qPCR cell counts for fungal phylotypes targeted by mold specific qPCR**. Click here for file

Additional file 6 **Table S5: Statistical pair-wise comparison of nucITS clone libraries from settled dust samples**. Click here for file

Additional file 7 **Table S6: List of performed qPCR assays and targeted species**. Click here for file

Additional file 8 **Table S7: Summary of analysed samples and applied methods**. Click here for file
