# Supplementary material for: Molecular profiling of fungal communities in moisture damaged buildings before and after remediation - a comparison of culture-dependent and culture-independent methods
Source: BMC Microbiol. 2011 Oct 21;11:235. doi: 10.1186/1471-2180-11-235 (PMC3206440; doi:10.1186/1471-2180-11-235)
Supplement: Additional file 1 — Fig. S1: Rarefaction curves for the analysed nucITS clone libraries. [file 1471-2180-11-235-S1.PDF]

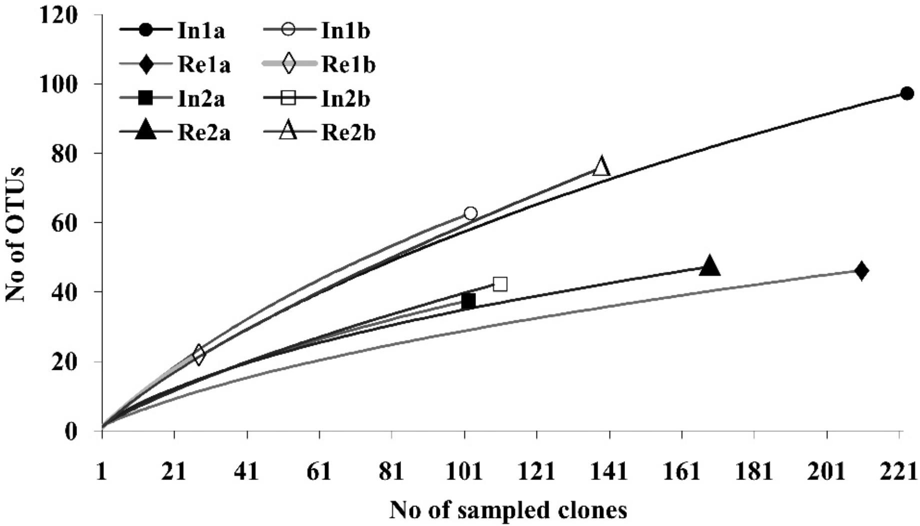

Fig. S1. Rarefaction analysis of ITS clone libraries constructed from settled dust samples at 1% OTU distance. The solid symbols denote libraries constructed from samples taken at time point a (before renovation of the index buildings), the open symbols denote samples taken at time point b (after renovation of the index buildings). Circle and square: index buildings, diamond and triangle: reference buildings.
