# Supplementary material for: Molecular profiling of fungal communities in moisture damaged buildings before and after remediation - a comparison of culture-dependent and culture-independent methods
Source: BMC Microbiol. 2011 Oct 21;11:235. doi: 10.1186/1471-2180-11-235 (PMC3206440; doi:10.1186/1471-2180-11-235)
Supplement: Additional file 2 — Table S1: Phylogenetic description, nearest database relative and frequency of detection of fungal molecular OTUs and isolated strains recovered from dust and water damaged building material. [file 1471-2180-11-235-S2.PDF]

Table S1. Detection frequencies and annotation details of NucITS clones and cultivated isolates from house dust samples.

Table S7. Phylogenetic description, nearest database relative and frequency of detection of fungal molecular OTUs and isolated strains recovered from dust and water damaged building materials.

Phylotype name	rDNA tree cluster*/ accession	Nearest Blast hit: Organism	Nearest Blast hit: Accession	Nearest Blast hit: Sim %	Final annotation **	Occurrence in dust and material samples ***: Location 1: In1a	Re1a	In1b	Re1b	BM-1	Location 2: In2a	Re2a	In2b	Re2b	BM-2
	Cluster* 1 - Eurotiomycetes					10	2	0	1		0	2	0	1	0
BF-OTU468	FR682211	Ascomycete sp.	AM292201	87.0	UA	1									
BF-OTU576	FR682319	*Capronia villosa*	AF050261	84.0	Eu				1						
BF-OTU418	FR682161	*Capronia villosa*	AF050261	84.0	Eu	1									
BF-OTU456	FR682199	*Cladonia furcata*	EU266080	98.0	Lc §	2									
BF-OTU408	FR682151	*Cladophialophora minutissima*	EF016382	81.0	Eu	1									
BF-OTU410	FR682153	*Cladophialophora minutissima*	EF016382	99.3	*Cladophialophora minutissima*	2									
BF-OTU695	FR682438	*Cladophialophora* sp.	EF016382	92.0	Eu §	2						1			
BF132	FR718472	*Exophiala xenobiotica*	DQ182590	100.0	*Exophiala sp.*										C
BF-OTU511	FR682254	Fungal sp.	AY843097	92.7	UA		2								
BF133	FR718473	*Rhinocladiella atrovirens*	AY618683	98.9	*Rhinocladiella atrovirens*										C
BF-OTU698	FR682441	Uncultured fungus	AJ582963	83.3	Eu							1			
BF-OTU438	FR682181	Uncultured fungus	DQ309126	83.5	Eu	1									
BF-OTU597	FR682340	Uncultured fungus	AM260819	96.5	Eu §									1	
	Cluster 2 - Eurotiomycetes					1	0	0	0		0	1	0	3	0
BF-OTU586	FR682329	No full length match			UF									1	
BF-OTU469	FR682212	No match			UF	1									
BF-OTU700	FR682443	*Phialocephala fluminis*	AF486124	82.1	Le							1			
BF-OTU585	FR682328	*Phialophora sessilis*	AY857542	99.5	*Phialophora sessilis*									1	
BF-OTU589	FR682332	Uncultured fungus	AJ582963	83.1	Eu									1	
	Cluster 3 - Unknown					0	2	0	1		1	3	0	2	0
BF-OTU612	FR682355	Fungal sp.	AY843052	99.6	Le §									1	
BF-OTU496	FR682239	Leaf litter ascomycete	AF502889	96.2	Eu		2								
BF-OTU582	FR682325	*Phaeococcomyces chersonesos*	AJ507323	90.3	Eu				1						
BF-OTU630	FR682373	*Phaeococcomyces chersonesos*	AJ507323	90.3	Eu									1	
BF-OTU676	FR682419	*Phaeococcomyces chersonesos*	AJ507323	90.9	Eu §						1				
BF-OTU697	FR682440	*Phialocephala fluminis*	AF486124	86.1	Le							1			
BF-OTU687	FR682430	Uncultured Peltigerales	FJ554149	98.6	Lc §							2			
	Cluster 4 - Unknown					0	0	1	0		0	2	0	0	0
BF-OTU686	FR682429	*Verrucaria* sp.	FJ664883	82.0	Eu							2			
BF-OTU521	FR682264	*Capronia villosa*	AF050261	82.0	Eu			1							
	Cluster 5 - Dothideomycetes					8	0	0	0		0	0	0	0	0
BF-OTU455	FR682198	*Botryosphaeria dothidea*	DQ008327	79.2	Do	2									
BF-OTU443	FR682186	Fungal sp.	AY843079	81.8	UA	1									
BF-OTU462	FR682205	Fungal sp.	AY843079	80.8	UA	4									
BF-OTU419	FR682162	*Mycocalicium subtile*	AF225440	76.3	Eu	1									
	Cluster 6 - Dothideomycetes					1	0	1	0		2	0	1	1	0
BF-OTU484	FR682227	*Alternaria citri*	AY154705	100.0	*Alternaria citri*	1							1	1	
BF-OTU541	FR682284	*Bipolaris sorokiniana*	EF187908	99.6	*Bipolaris sorokiniana*			1							

Table S1. Detection frequencies and annotation details of NucITS clones and cultivated isolates from house dust samples.

Phylotype name	rDNA tree cluster*/ accession	Nearest Blast hit			Final annotation **	Occurrence in dust and material samples ***									
						Location 1					Location 2				
		Organism	Accession	Sim %		In1a	Re1a	In1b	Re1b	BM-1	In2a	Re2a	In2b	Re2b	BM-2
BF-OTU662	FR682405	*Phaeococcomyces nigricans*	AF050278	80.7	Eu §						2				
	Cluster 7 - Unknown					1	0	0	0		0	0	0	0	0
BF-OTU440	FR682183	*Mycosphaerella swartii*	DQ923536	84.0	Do	1									
	Cluster 8 - Leotiomycetes					0	0	0	0		0	1	0	1	0
BF-OTU692	FR682435	*Mollisia fusca*	AY259138	98.8	*Mollisia fusca*							1			
BF-OTU610	FR682353	Melanized limestone ascomycete	AY559378	92.3	Do									1	
	Cluster 9 - Unknown					0	0	0	0		0	0	0	1	0
BF-OTU628	FR682371	Uncultured ascomycete isolate	AY969391	93.4	Le									1	
	Cluster 10 - Unknown					3	0	2	0		0	0	0	0	0
BF-OTU409	FR682152	*Colletogloeopsis blakelyi*	DQ923526	82.7	Do §	3		2							
	Cluster 11 - Eurotiomycetes					1	0	1	0		0	0	0	0	0
BF-OTU454	FR682197	*Phaeococcomyces nigricans*	AY843154	99.4	*Phaeococcomyces nigricans*	1		1							
	Cluster 12 - Eurotiomycetes/Leotiomycetes					3	0	2	0	134	49	5	59	8	0
BF-OTU721	FR682463	*Aspergillus conicus*	AY373865	96.8	*Aspergillus* sp.					1					
BF-OTU716	FR682458	*Aspergillus conicus*	EF652039	98.0	*Aspergillus conicus*					2					
BF-OTU552	FR682295	*Aspergillus conicus*	EF652039	98.0	*Aspergillus conicus*			1		28			1	1	
BF-OTU724	FR682466	*Aspergillus conicus*	EF652039	98.9	*Aspergillus conicus*					1					
BF-OTU719	FR682461	*Aspergillus conicus*	AY373865	99.0	*Aspergillus conicus*					44					
BF-OTU717, BF043	FR682459, FR718462	*Aspergillus conicus*	EF652039	99.0	*Aspergillus conicus*					C, 1					
BF-OTU704	FR682447	*Aspergillus conicus*	AY373864	99.0	*Aspergillus conicus*					1					
BF-OTU722	FR682464	*Aspergillus conicus*	EF652039	97.8	*Aspergillus* sp.					1					
BF051	FR718464	*Aspergillus nidulans*	AF455505	100.0	*Aspergillus nidulans*					C					
BF-OTU632	FR682375	*Aspergillus ochraceus*	AY609210	100.0	*Aspergillus ochraceus*								1		
BF030	FR718457	*Aspergillus sydowii*	AY373868	99.6	*Aspergillus sydowii*					C					
BF-OTU710	FR682453	*Eupenicillium javanicum*	AY373921	89.0	Eu					1					
BF-OTU711	FR682454	*Eupenicillium javanicum*	U18358	89.9	Eu					1					
BF-OTU713	FR682456	*Eupenicillium javanicum*	U18358	90.1	Eu					23					
BF-OTU709	FR682452	*Eupenicillium javanicum*	U18358	90.2	Eu					3					
BF-OTU712	FR682455	*Eupenicillium javanicum*	U18358	90.3	Eu					23					
BF-OTU649	FR682392	*Eurotium amstelodami*	AF455464	99.4	*Eurotium* sp.								1		
BF-OTU648	FR682391	*Eurotium niveoglaucum*	AF459728	100.0	*Eurotium* sp.					1			1		
BF-OTU472	FR682215	*Geomyces pannorum*	DQ189229	99.8	*Geomyces pannorum*	1									
BF001	FR718449	*Paecilomyces divaricatus*	AY7373851	99.8	*Paecilomyces divaricatus*					C					
BF029	FR718456	*Paecilomyces variotii*	AF033395	100.0	*Paecilomyces variotii*					C					
BF-OTU532, BF167	FR682275, FR718486	*Penicillium brevicompactum*	AY373898	99,8-100	*Penicillium* sp.	2		1							C
BF027	FR718455	*Penicillium canescens*	AF033493	99.8	*Penicillium* sp.					C					
BF-OTU679, BF115	FR682422, FR718468	*Penicillium chrysogenum*	AF034449	99,8-100	*Penicillium* sp.	C				C, 1	C, 36	C, 5	46	1	C
BF006	FR718452	*Penicillium citreonigrum*	AF033456	100.0	*Penicillium citreonigrum*					C					

Table S1. Detection frequencies and annotation details of NucITS clones and cultivated isolates from house dust samples.

Phylotype name	rDNA tree cluster*/ accession	Nearest Blast hit			Final annotation **	Occurrence in dust and material samples ***									
		Organism	Accession	Sim %		Location 1					Location 2				
						In1a	Re1a	In1b	Re1b	BM-1	In2a	Re2a	In2b	Re2b	BM-2
BF-OTU651, BF25	FR682394, FR718453	*Penicillium commune*	AF236103	99.8	*Penicillium* sp.					C	13		9		
BF-OTU624, BF037	FR682367, FR718459	*Penicillium corylophilum*	AF034457	99,8-100	*Penicillium corylophilum*					C, 1				1	C
BF-OTU608	FR682351	*Penicillium glabrum*	AF033407	99.8	*Penicillium* sp.									1	
BF-OTU587	FR682330	*Penicillium roquefortii*	AY373929	99.8	*Penicillium* sp.									3	
BF026	FR718454	*Penicillium* sp.	AB297800	92.0	Le					C					
BF-OTU723, BF005	FR682465, FR718451	*Penicillium* sp.	FJ379809	99.1	*Penicillium* sp.					C, 1					
BF042	FR718461	*Penicillium spinulosum*	GU566252	99.8	*Penicillium* sp.					C					
BF-OTU602	FR682345	*Pleopsidium chlorophanum*	AY853384	82.6	Lc									1	
	Cluster 13 - Lecanoromycetes					3	2	0	0		0	0	0	1	0
BF-OTU416	FR682159	*Hypogymnia physodes*	AF058036	99.1	*Hypogymnia physodes*	2								1	
BF-OTU505	FR682248	*Parmelia sulcata*	AF410839	100.0	*Parmelia sulcata*		2								
BF-OTU467	FR682210	*Parmeliopsis ambigua*	AF451764	99.8	*Parmeliopsis ambigua*	1									
	Cluster 14 - Leotiomycetes					5	2	0	0		4	3	1	7	0
BF-OTU504	FR682247	*Lachnum bicolor*	AJ430394	99.8	*Lachnum bicolor*		1								
BF-OTU688	FR682431	*Meria laricis*	U92299	99.8	*Meria laricis*							2			
BF-OTU499	FR682242	*Monilinia fructigena*	EF207427	100.0	*Monilinia fructigena*		1								
BF-OTU627	FR682370	*Mycocalicium victoriae*	AJ312123	94.8	Eu §	1								1	
BF-OTU411	FR682154	*Sclerotinia sclerotiorum*	AF455523	99.8	Le	4					4	1	1	6	
	Cluster 15 - Lecanoromycetes					1	1	0	0		0	0	1	0	0
BF-OTU631	FR682374	*Lecanora saligna*	AF189716	85.0	Lc §								1		
BF-OTU510	FR682253	*Miriquidica garovaglii*	EU075538	85.8	Lc §	1	1								
	Cluster 16 - Lecanoromycetes					0	1	0	0		0	1	0	1	0
BF-OTU619	FR682362	*Lecidella carpathica*	DQ534471	75.8	Lc									1	
BF-OTU486	FR682229	Uncultured fungus	AJ875379	77.5	UF §		1					1			
	Cluster 17 - Dothideomycetes					24	14	13	1		3	4	8	2	0
BF-OTU483	FR682226	*Ampelomyces* sp.	AF035783	100.0	*Ampelomyces* sp.		3								
BF-OTU653	FR682396	*Ascochyta* sp.	AY305377	99.6	*Ascochyta* sp.								1		
BF-OTU671	FR682414	*Epicoccum nigrum*	AF455455	99.8	*Epicoccum nigrum*	6					1				
BF-OTU681	FR682424	Fungal cont. of *Leptosphaeria* sp.	AJ002205	80.8	Pe							1			
BF-OTU555	FR682298	Fungal sp.	AM231348	99.6	Do §			1							
BF-OTU497, BF048	FR682240, FR718463	*Leptosphaerulina chartarum*	DQ384571	99,5-100	*Leptosphaerulina chartarum*	10	6	4		C	2				
BF-OTU422	FR682165	*Leptosphaerulina trifolii*	AY831558	99.8	Do	2									
BF-OTU458	FR682201	*Lophium mytilinum*	EF596819	99.6	*Lophium mytilinum*	1									
BF-OTU574	FR682317	*Phaeosphaeria avenaria*	U77359	95.5	*Phaeosphaeria* sp.				1						
BF-OTU559	FR682302	*Phaeosphaeria* sp.	AY345346	93.0	Do §			1							
BF-OTU546	FR682289	Phaeosphaeriaceae sp.	AY465459	96.5	Do §			1							
BF-OTU507	FR682250	*Phaeosphaeriopsis amblyspora*	AY188993	89.7	Do §	1	1								
BF-OTU642	FR682385	*Phoma exigua*	AJ608976	99.0	*Phoma exigua*								1		

Table S1. Detection frequencies and annotation details of NucITS clones and cultivated isolates from house dust samples.

Phylotype name	rDNA tree cluster*/ accession	Nearest Blast hit			Final annotation **	Occurrence in dust and material samples ***									
						Location 1					Location 2				
		Organism	Accession	Sim %		In1a	Re1a	In1b	Re1b	BM-1	In2a	Re2a	In2b	Re2b	BM-2
BF-OTU564	FR682307	*Phoma exigua*	AJ608976	99.6	*Phoma* sp.			1					1		
BF-OTU528, BF153	FR682271, FR718481	*Phoma herbarum*	AY293800	100.0	*Phoma herbarum*			1						1	C
BF-OTU431, BF053	FR682174, FR718466	*Phoma herbarum*	AY337712	100.0	*Phoma herbarum*	1				C					
BF-OTU413	FR682156	*Phoma macrostoma*	DQ474114	99.8	*Phoma* sp.	3	1						2		
BF-OTU693	FR682436	*Phoma* sp.	EF423518	99.0	*Phoma* sp.							3			
BF-OTU534	FR682277	*Phoma* sp.	DQ344033	99.4	*Phoma* sp.			3							
BF-OTU654, BF143	FR682397, FR718477	*Phoma* sp.	AY465466	99,5-100	*Phoma* sp.		2						1		C
BF-OTU527, BF168	FR682270, FR718487	*Phoma* sp.	AF218789	99,8-100	*Phoma* sp.			1							C
BF-OTU641	FR682384	*Pleiochaeta setosa*	EU167563	93.3	AsIs								2		
BF-OTU592	FR682335	Uncultured fungus isolate	DQ093786	86.4	UF									1	
BF-OTU494	FR682237	Uncultured soil fungus clone	DQ420828	96.2	Pe		1								
	Cluster 18 - Dothideomycetes					30	13	4	6	3	6	9	4	24	0
BF-OTU708	FR682451	*Amorphotheca resinae*		81.0	UF					3					
BF-OTU459	FR682202	Ascomycete sp.	EF373580	85.5	Do	1									
BF-OTU572	FR682315	*Aureobasidium pullulans*	DQ640766	99.8	*Aureobasidium pullulans*	1			1		1	3		2	
BF-OTU616	FR682359	*Aureobasidium pullulans*	AY213639	99.8	*Aureobasidium pullulans*									3	
BF-OTU415	FR682158	*Aureobasidium pullulans*	AM160630	100.0	*Aureobasidium pullulans*	11	5	3	1		2	2	1	16	
BF-OTU609	FR682352	*Dothichiza pityophila*	AJ244242	80.0	Do									1	
BF-OTU498	FR682241	Ectomycorrhizal isolate	AY345353	97.6	Le		1								
BF-OTU519	FR682262	*Elsinoe ampelina*	AY826762	84.2	Do			1							
BF-OTU463	FR682206	Fungal sp.	AY843105	92.9	UA §	2									
BF-OTU476	FR682219	*Hormonema* aff. *prunorum*	AY188367	98.0	*Hormonema* sp.	3	6		3		2	1			
BF-OTU426	FR682169	*Hormonema carpetanum*	AY616200	99.0	Do	2									
BF-OTU573, BF125	FR682316, FR718470	*Hormonema dematioides*	AJ278927	99,1-100	*Hormonema dematioides*	1			1				1	1	C
BF-OTU417	FR682160	*Hypocenomyce scalaris*	DQ782852	97.3	Lc	1									
BF-OTU445	FR682188	*Micarea lapillicola*	AY756479	86.3	Lc	2									
BF-OTU677	FR682420	*Mycosphaerella africana*	AY626981	98.2	*Mycosphaerella* sp.						1				
BF-OTU406	FR682149	*Mycosphaerella parva*	AY626980	84.0	Do	3									
BF-OTU690	FR682433	*Mycosphaerella* sp.	DQ458904	94.5	Do §							3			
BF-OTU430	FR682173	No full length match			UF §	2									
BF-OTU513	FR682256	*Pringsheimia smilacis*	AJ244257	98.1	*Pringsheimia smilacis*		1								
BF-OTU407	FR682150	*Sawadaea tulasnei*	AB193361	99.4	*Sawadaea tulasnei*	1									
BF-OTU622	FR682365	*Sphaerulina eucalypti*	AY293060	79.5	Do §									1	
BF-OTU638	FR682381	*Trimmatostroma abietis*	AY128697	99.8	*Trimmatostroma* sp.								2		
	Cluster 19 - Dothideomycetes					5	1	0	0		0	0	13	0	0
BF-OTU427	FR682170	Fungal sp.	AY843079	84.6	Do	1									
BF-OTU412	FR682155	Fungal sp.	AY843191	87.7	Do §	1							2		

Table S1. Detection frequencies and annotation details of NucITS clones and cultivated isolates from house dust samples.

Phylotype name	rDNA tree cluster*/ accession	Nearest Blast hit: Organism	Nearest Blast hit: Accession	Nearest Blast hit: Sim %	Final annotation **	Occurrence in dust and material samples ***: Location 1: In1a	Re1a	In1b	Re1b	BM-1	Location 2: In2a	Re2a	In2b	Re2b	BM-2
BF-OTU508	FR682251	Fungal sp.	AY843115	98.6	Eu §		1								
BF-OTU447	FR682190	Fungal sp.	AY843191	99.0	Do §	1									
BF-OTU637	FR682380	*Rhizoctonia bataticola*	DQ222241	76.0	Ag								11		
BF-OTU414	FR682157	Unknown dothideomycete	AY560007	100.0	Do	2									
	Cluster 20 - Dothideomycetes					41	138	11	0		5	3	10	9	0
BF-OTU493	FR682236	*Cladosporium cladosporioides*	AY361968	99.6	*Cladosporium* sp.		1								
BF-OTU540	FR682283	*Cladosporium cladosporioides*	AF455525	100.0	*Cladosporium* sp.			1				2	2	6	
BF-OTU405, BF141	FR682148, FR718476	*Cladosporium cladosporioides*	AF393691	100.0	*Cladosporium* sp.	3	15	4			4		3	1	C
BF150	FR718479	*Cladosporium cladosporioides*	DQ426533	100.0	*Cladosporium* sp.					C					
BF-OTU491	FR682234	*Cladosporium herbarum*	AY251078	99.0	*Cladosporium* sp.		1								
BF-OTU425, BF060	FR682168, FR718467	*Cladosporium herbarum*	AF393712	100.0	*Cladosporium* sp.	15	1	4		C	1		5	1	
BF-OTU503, BF129	FR682246, FR718471	*Cladosporium* sp.	AJ300336	99,6-100	*Cladosporium* sp.		1								C
BF-OTU478	FR682221	*Cladosporium sphaerospermum*	AF455481	100.0	*Cladosporium* sp.	1	115	1							
BF-OTU613	FR682356	*Eudarluca caricis*	AY607017	99.6	*Cladosporium* sp.									1	
BF-OTU433	FR682176	Fungal sp.	AY843079	84.9	Lc	1									
BF-OTU446	FR682189	Fungal sp.	AY843182	99.4	Do §	2	1	1							
BF-OTU423	FR682166	*Phaeotheca fissurella*	AJ244255	86.4	Do §	10	3					1			
BF-OTU450	FR682193	*Phaeothecoidea proteae*	EU707898	96.8	*Phaeothecoidea* sp.	7									
BF-OTU473	FR682216	*Roccella peruensis*	AJ634026	70.3	Art	1									
BF-OTU453	FR682196	*Sarcinomyces crustaceus*	AJ244258	99.0	*Sarcinomyces crustaceus*	1									
	Cluster 21 - Dothideomycetes					1	0	1	0	3	0	0	0	1	0
BF-OTU583	FR682326	*Fusicladium fagi*	EU035431	99.8	*Cladosporium nigrellum*									1	
BF-OTU707	FR682450	*Mycosphaerella flexuosa*	DQ302958	83.4	Do					2					
BF-OTU706	FR682449	*Mycosphaerella flexuosa*	DQ302958	83.4	Do					1					
BF-OTU449	FR682192	*Venturia ditricha*	EU035456	99.4	*Venturia* sp.	1									
BF-OTU530	FR682273	*Venturia tremulae*	EU035475	98.6	*Venturia* sp.			1							
	Cluster 22 - Sordariomycetes					1	0	1	1		0	2	0	2	0
BF-OTU621	FR682364	*Cadophora luteo-olivacea*	GQ214536	99.7	So									1	
BF-OTU516	FR682259	*Chaenothecopsis haematopus*	AY795861	98.1	*Chaenothecopsis haematopus*			1							
BF-OTU699	FR682442	*Helgardia* sp.	AM262430	100.0	*Helgardia* sp.							1			
BF-OTU457	FR682200	*Holwaya mucida*	DQ257357	88.0	Le	1									
BF134	FR718474	*Cadophora* sp.	AY371513	100.0	Le										C
BF-OTU598	FR682341	*Mycocalicium victoriae*	AJ312123	88.2	Eu									1	
BF-OTU568	FR682311	*Mycocalicium victoriae*	AJ312123	94.9	Eu				1						
BF-OTU703	FR682446	*Mycocalicium victoriae*	AJ312123	90.8	Eu							1			
	Cluster 23 - Sordariomycetes					4	8	2	0	1	1	15	2	23	0
BF052	FR718465	*Acremonium alternatum*	AM176679	86.9	So					C					
BF-OTU623	FR682366	*Acremonium alternatum*	AM176679	89.4	So									1	
BF-OTU650	FR682393	*Acremonium alternatum*	U57674	91.3	So								1		

Table S1. Detection frequencies and annotation details of NucITS clones and cultivated isolates from house dust samples.

Phylotype name	rDNA tree cluster*/ accession	Nearest Blast hit: Organism	Nearest Blast hit: Accession	Nearest Blast hit: Sim %	Final annotation **	Occurrence in dust and material samples ***: Location 1: In1a	Re1a	In1b	Re1b	BM-1	Location 2: In2a	Re2a	In2b	Re2b	BM-2
BF-OTU477	FR682220	*Acremonium alternatum*	AM176679	100.0	*Acremonium* sp.		2							13	
BF-OTU618	FR682361	*Acremonium cyanophagus*	DQ393594	83.4	So									1	
BF-OTU480	FR682223	*Cephalosporium* sp.	AM176722	99.8	So		3								
BF-OTU464	FR682207	*Chaetomium globosum*	DQ003217	99.8	*Chaetomium globosum*	1									
BF-OTU531	FR682274	*Colletotrichum linicola*	AB046609	99.5	*Colletotrichum* sp.			1							
BF-OTU500	FR682243	*Cryptodiaporthe* cf. *Hystrix*	EU255027	99.8	*Cryptodiaporthe* sp.		1								
BF-OTU615	FR682358	*Cryptodiaporthe salicella*	DQ323529	98.9	*Cryptodiaporthe salicella*									1	
BF-OTU565	FR682308	*Diatrypella pulvinata*	AJ302443	99.8	*Diatrypella pulvinata*			1							
BF-OTU421	FR682164	*Fusarium oxysporum*	AF538623	99.8	*Fusarium* sp.	3								1	
BF-OTU684	FR682427	*Fusarium oxysporum*	DQ535184	99,6-100	*Fusarium oxysporum*							13			
BF135	FR718475	*Lecythophora hoffmannii*	AY805566	100.0	*Lecythophora hoffmannii*										C
BF-OTU487	FR682230	*Microdochium nivale*	EF187912	99.8	*Microdochium nivale*		1								
BF-OTU512	FR682255	*Myrothecium atroviride*	AJ302002	100.0	*Myrothecium* sp.		1								
BF-OTU675	FR682418	No full length match			UF §						1	1			
BF-OTU605	FR682348	*Nodulisporium* sp.	EF600033	99.8	*Nodulisporium* sp.									1	
BF-OTU606	FR682349	*Paecilomyces major*	DQ243696	97.7	*Paecilomyces* sp.									2	
BF-OTU705, BF039	FR682448, FR718460	*Phialophora* sp.	AY618679	99,2-99,3	*Phialophora* sp.					C, 5					
BF-OTU600	FR682343	*Sordariomycete* sp.	DQ227290	89.2	So									2	
BF-OTU718	FR682460	*Stilbella byssiseda*	AF335453	83.5	So					1					
BF002	FR718450	*Thielavia hyalocarpa*	AJ271583	98.0	So					C					
BF152	FR718480	*Trichoderma atroviride*	EF417482	99.8	*Trichoderma* sp.										C
BF124	FR718469	*Trichoderma citrinoviride*	AJ230663	100.0	*Trichoderma* sp.										C
BF-OTU601	FR682344	*Trichoderma harzianum*	AJ230664	99.8	*Trichoderma* sp.							1		1	
BF-OTU636	FR682379	*Trichoderma koningii*	AF218790	99.8	*Trichoderma* sp.								1		
	Cluster 24 - Taphrinomycetes					0	0	0	0		0	1	0	0	0
BF-OTU689	FR682432	*Taphrina ulmi*	AF492123	90.3	Ta							1			
	Cluster 25 - Saccharomycetes					3	0	3	0		0	73	0	7	0
BF-OTU526	FR682269	*Candida famata*	AM992913	99.8	*Candida* sp.	3		2				1		4	
BF-OTU593	FR682336	*Candida zeylanoides*	AB278160	99.3	*Candida zeylanoides*									1	
BF-OTU694	FR682437	*Saccharomyces cerevisiae*	AY939814	99.3	*Saccharomyces cerevisiae*							1			
BF-OTU550	FR682293	*Saccharomyces cerevisiae*	D89886	99.8	*Saccharomyces cerevisiae*			1				46		1	
BF-OTU629	FR682372	*Saccharomyces cerevisiae*	U53879	99.6	*Saccharomyces cerevisiae*							25		1	
	Cluster 26 - Unknown					1	0	0	0		0	0	0	0	0
BF-OTU448	FR682191	No match			UF	1									
	Cluster 27 - Tremellomycetes					1	11	5	0		2	2	0	11	0
BF-OTU599	FR682342	*Cryptococcus carnescens*	AB105432	99.8	*Cryptococcus carnescens*									5	
BF-OTU481	FR682224	*Cryptococcus carnescens*	EU149786	99.8	*Cryptococcus carnescens*		7								
BF-OTU479	FR682222	*Cryptococcus festucosus*	AY633979	99.4	*Cryptococcus festucosus*	1	1				2				
BF-OTU485	FR682228	*Cryptococcus foliicola*	AY557600	97.4	*Cryptococcus* sp.		2								
BF-OTU596	FR682339	*Cryptococcus* sp.	DQ317359	98.8	*Cryptococcus* sp.									1	
BF-OTU520	FR682263	*Cryptococcus tephrensis*	DQ000318	98.3	*Cryptococcus* sp.			2				2		2	

Table S1. Detection frequencies and annotation details of NucITS clones and cultivated isolates from house dust samples.

Phylotype name	rDNA tree cluster*/ accession	Nearest Blast hit			Final annotation **	Occurrence in dust and material samples ***									
						Location 1					Location 2				
		Organism	Accession	Sim %		In1a	Re1a	In1b	Re1b	BM-1	In2a	Re2a	In2b	Re2b	BM-2
BF-OTU607	FR682350	*Cryptococcus* sp.	DQ317359	98.2	*Cryptococcus* sp.									1	
BF-OTU557	FR682300	*Cryptococcus tephrensis*	DQ000318	97.9	*Cryptococcus* sp.			1							
BF-OTU489	FR682232	*Cryptococcus victoriae*	AF444469	98.8	*Cryptococcus victoriae*		1							1	
BF-OTU515	FR682258	*Cryptococcus victoriae*	AF444447	99.4	*Cryptococcus victoriae*			2						1	
	Cluster 28 - Tremellomycetes					4	1	1	1		1	0	0	0	0
BF-OTU451	FR682194	*Cryptococcus hungaricus*	AF272664	97.7	*Cryptococcus* sp.	1									
BF-OTU471	FR682214	*Cryptococcus hungaricus*	AF444379	99.3	*Dioszegia hungarica*	1									
BF-OTU577	FR682320	*Cryptococcus rajasthanensis*	AM262325	94.2	Tr				1						
BF-OTU403	FR682146	*Cryptococcus* sp.	AF444332	75.8	Tr	1									
BF-OTU506	FR682249	*Fellomyces fuzhouensis*	AF444401	75.8	Tr		1								
BF-OTU465	FR682208	*Tremella giraffa*	AF042453	86.3	Tr	1									
BF-OTU659	FR682402	Tremellales sp.	EF060541	97.8	Tr						1				
BF-OTU545	FR682288	Uncultured basidiomycete	AY969511	80.6	Tr			1							
	Cluster 29 - Agaricomycetes					1	0	1	0		4	0	0	0	0
BF-OTU558	FR682301	*Collybia dryophila*	AY521249	98.7	*Gymnopus* sp.			1							
BF-OTU668	FR682411	*Gymnopus dryophilus*	AY256690	99.6	*Gymnopus dryophilus*						1				
BF-OTU474	FR682217	*Mycena galericulata*	DQ404392	99.7	*Mycena galericulata*	1									
BF-OTU666	FR682409	*Mycena luteopallens*	AB512313	89.5	Ag §						3				
	Cluster 30 - Agaricomycetes					1	0	2	0		1	0	1	2	0
BF-OTU667	FR682410	*Armillaria ostoyae*	AY213552	99.5	*Armillaria ostoyae*						1				
BF-OTU647	FR682390	*Cylindrobasidium evolvens*	AY618227	99.6	Ag								1		
BF-OTU543	FR682286	*Flammulina velutipes*	AF036928	99.3	*Flammulina velutipes*			2							
BF-OTU400	FR682143	*Pleurotus salmoneostramineus*	AY265843	65.8	Ag §	1									
BF-OTU620	FR682363	*Stephanospora caroticolor*	AJ419224	81.0	Ag									2	
	Cluster 31 - Unknown					0	0	0	0		1	0	0	0	0
BF-OTU663	FR682406	*Rhizoctonia* sp.	AY927338	68.7	Ag §						1				
	Cluster 32 - Agaricomycetes					0	0	0	0		1	0	0	1	0
BF-OTU672	FR682415	*Typhula variabilis*	AB267394	97.2	Ag §						1				
BF-OTU591	FR682334	*Typhula variabilis*	AB267394	96.9	Ag									1	
	Cluster 33 - Microbotryomycetes					0	0	1	0		0	1	3	0	0
BF-OTU691	FR682434	Antarctic yeast	AY040664	99.7	Mi §							1			
BF-OTU551	FR682294	*Leucosporidium scottii*	AM117830	99.8	*Leucosporidium* sp.			1							
BF155	FR718482	*Rhodosporidium lusitaniae*	AY015430	94.4	Mi										C
BF-OTU644	FR682387	*Rhodosporidium lusitaniae*	AJ853459	99.8	*Rhodosporidium lusitaniae*								1		
BF145	FR718478	*Sporodiobolus salmonicolor*	AY015434	100.0	*Sporodiobolus salmonicolor*										C
BF-OTU656	FR682399	*Sporobolomyces jilinensis*	AY364838	97.7	*Sporobolomyces* sp.								1		
BF-OTU639	FR682382	*Sporobolomyces ruberrimus*	AY015436	100.0	*Sporobolomyces* sp.								1		
	Cluster 34 - Agaricomycetes					0	0	0	2	1	0	0	1	1	0
BF-OTU595	FR682338	*Armillaria novae-zelandiae*	AF394919	82.0	Ag									1	
BF-OTU652	FR682395	*Auriscalpium vulgare*	AF454430	99.5	*Auriscalpium vulgare*								1		
BF-OTU714	FR682457	*Eupenicillium javanicum*	U18358	89.0	Eu					1					
BF-OTU571	FR682314	*Rhizoctonia cerealis*	AF063019	99.5	Ag				2						

Table S1. Detection frequencies and annotation details of NucITS clones and cultivated isolates from house dust samples.

Phylotype name	rDNA tree cluster*/ accession	Nearest Blast hit: Organism	Nearest Blast hit: Accession	Nearest Blast hit: Sim %	Final annotation **	Occurrence in dust and material samples ***: Location 1: In1a	Re1a	In1b	Re1b	BM-1	Location 2: In2a	Re2a	In2b	Re2b	BM-2
	Cluster 35 - Tremellomycetes					0	0	0	1		1	0	0	1	0
BF-OTU588	FR682331	*Phlebiopsis gigantea*	AF087487	99.5	*Phlebiopsis gigantea*									1	
BF-OTU570	FR682313	*Cryptococcus albidosimilis*	AF145331	100.0	*Cryptococcus* sp.				1		1				
	Cluster 36 - Tremellomycetes					0	0	0	0		0	3	0	0	0
BF-OTU682	FR682425	*Filobasidium uniguttulatum*	DQ668348	99.8	*Filobasidium uniguttulatum*							3			
	Cluster 37 - Microbotryomycetes					0	2	0	0		0	6	1	0	0
BF-OTU488	FR682231	*Rhodotorula glutinis*	AM160642	99.8	Mi		2								
BF-OTU646, BF165	FR682389, FR718484	*Rhodotorula mucilaginosa*	DQ386306	100.0	*Rhodotorula mucilaginosa*							6	1		C
	Cluster 38 - Tremellomycetes					7	0	7	0		3	14	8	7	0
BF-OTU561, BF166	FR682304, FR718485	*Cryptococcus adeliensis*	AF145328	99,7-100	*Cryptococcus adeliensis*			1				1	3	1	C
BF-OTU590	FR682333	*Cryptococcus albidus*	AF444378	99.7	*Cryptococcus albidus*	2								1	
BF-OTU460	FR682203	*Cryptococcus friedmannii*	AF145322	97.2	*Cryptococcus* sp.	2									
BF-OTU640	FR682383	*Cryptococcus friedmannii*	AF145322	98.3	*Cryptococcus* sp.	1							1		
BF-OTU604	FR682347	*Cryptococcus magnus*	AF190008	99.2	*Cryptococcus magnus*									1	
BF-OTU524	FR682267	*Cryptococcus magnus*	AF190009	99.8	*Cryptococcus magnus*			3			1			3	
BF-OTU635	FR682378	*Cryptococcus oeirensis*	AY188362	99.5	*Cryptococcus oeirensis*								1		
BF-OTU669	FR682412	*Cryptococcus oeirensis*	AF444364	99.5	*Cryptococcus oeirensis*						2				
BF-OTU643	FR682386	*Cryptococcus saitoi*	AF444372	99.7	*Cryptococcus saitoi*								1		
BF-OTU529	FR682272	*Cryptococcus wieringae*	AF444383	99.7	*Cryptococcus wieringae*	2		3				5	1		
BF-OTU633	FR682376	*Mrakia frigida*	AF144482	99.8	*Mrakia* sp.								1		
BF-OTU617	FR682360	*Mrakia gelida*	AF144485	100.0	*Mrakia* sp.							7		1	
BF-OTU702	FR682445	*Mrakia* sp.	AY038836	99.3	*Mrakia* sp.							1			
	Cluster 41 - Tremellomycetes					1	0	2	1		0	1	0	2	0
BF159	FR718483	*Cryptococcus skinneri*	AF444305	96.1	Tr										C
BF-OTU611	FR682354	*Cystofilobasidium capitatum*	AY052492	99.8	*Cystofilobasidium capitatum*	1								1	
BF-OTU542	FR682285	*Cystofilobas. infirmo-miniatum*	AF444400	100.0	*C. infirmo-miniatum*			1							
BF-OTU575	FR682318	*Cystofilobasidium macerans*	AF444317	99.8	*Cystofilobasidium macerans*				1						
BF-OTU562	FR682305	*Trichosporon mucoides*	AF455482	99.8	*Trichosporon* sp.			1				1		1	
	Cluster 42 - Agaricomycetes					1	0	0	0		0	0	2	0	0
BF-OTU634	FR682377	*Exidia japonica*	AF291274	93.9	Ag								2		
BF-OTU404	FR682147	*Exidia pithya*	AY805609	100.0	*Exidia pithya*	1									
	Cluster 43 - Microbotryomycetes					0	1	1	0		0	0	0	1	0
BF-OTU495	FR682238	*Exobasidium pachysporum*	AB180352	96.3	*Exobasidium* sp.		1								
BF-OTU614	FR682357	*Exobasidium woronichinii*	AB180361	95.8	*Exobasidium* sp.									1	
BF-OTU556	FR682299	*Rhodotorula phylloplana*	AB038131	99.0	*Rhodotorula* sp.			1							
	Cluster 44 - Basidiomycetes *incertae sedis*					9	1	16	5		1	9	2	10	0
BF-OTU678	FR682421	*Malassezia globosa*	AY743630	98.1	*Malassezia globosa*						1	1			
BF-OTU517	FR682260	*Malassezia globosa*	AY387134	98.7	*Malassezia globosa*			1							
BF-OTU537	FR682280	*Malassezia globosa*	AY743630	99.3	*Malassezia globosa*			2	2				1	1	
BF-OTU544	FR682287	*Malassezia globosa*	AY387136	99.6	*Malassezia globosa*			1						2	

Table S1. Detection frequencies and annotation details of NucITS clones and cultivated isolates from house dust samples.

Phylotype name	rDNA tree cluster*/ accession	Nearest Blast hit			Final annotation **	Occurrence in dust and material samples ***									
						Location 1					Location 2				
		Organism	Accession	Sim %		In1a	Re1a	In1b	Re1b	BM-1	In2a	Re2a	In2b	Re2b	BM-2
BF-OTU536	FR682279	*Malassezia restricta*	AJ437695	95.9	*Malassezia sp.*			2							
BF-OTU535	FR682278	*Malassezia restricta*	AJ437695	96.0	*Malassezia sp.*			1					1		
BF-OTU420	FR682163	*Malassezia restricta*	AJ437695	96.4	*Malassezia sp.*	1			1						
BF-OTU401	FR682144	*Malassezia restricta*	AJ437695	96.9	*Malassezia sp.*	2									
BF-OTU554	FR682297	*Malassezia restricta*	AY387145	99.3	*Malassezia restricta*	2		1				3		1	
BF-OTU522	FR682265	*Malassezia restricta*	AJ437695	99.9	*Malassezia restricta*	1		4	1			4		6	
BF-OTU429	FR682172	*Malassezia sympodialis*	AY743646	79.3	BaIs §	2	1	4							
BF-OTU432	FR682175	*Malassezia sympodialis*	AY743632	100.0	*Malassezia sympodialis*	1						1			
BF-OTU567	FR682310	Uncultured Malasseziales	AY612334	98.3	*Malassezia* sp.				1						
	Cluster 45 - Agaricomycetes					0	0	1	0		0	0	0	0	0
BF-OTU539	FR682282	*Tricholoma lascivum*	AY573542	99.8	*Tricholoma* sp.			1							
	Cluster 46 - Agaricomycetes					5	0	4	1		2	2	0	3	0
BF-OTU696	FR682439	*Calvatia turneri*	DQ112596	99.3	*Lycoperdon* sp.							1			
BF-OTU683	FR682426	*Coprinopsis stercorea*	AY461828	91.4	Ag							1			
BF-OTU594	FR682337	*Cortinarius aprinus*	AY669663	97.7	*Cortinarius* sp.									1	
BF-OTU514	FR682257	*Cortinarius armillatus*	DQ114744	100.0	*Cortinarius armillatus*			1							
BF-OTU658	FR682401	*Cortinarius pholideus*	AY669694	94.6	Ag						1				
BF-OTU461	FR682204	*Dermocybe semisanguinea*	DQ481909	99.2	*Dermocybe* sp.	1									
BF-OTU584	FR682327	*Handkea excipuliformis*	DQ112590	99.0	*Lycoperdon* sp.									1	
BF-OTU533	FR682276	*Hebeloma mesophaeum*	AB211272	99.6	*Hebeloma* sp.			1							
BF-OTU549	FR682292	*Hypholoma capnoides*	AY805610	99.8	*Hypholoma capnoides*			1						1	
BF-OTU470	FR682213	*Hypholoma sublateritium*	AY818349	99.8	*Hypholoma sublateritium*	2									
BF-OTU580	FR682323	*Lycoperdon pyriforme*	AY854075	100.0	*Lycoperdon pyriforme*				1		1				
BF-OTU444	FR682187	*Panaeolus* sp.	DQ403253	99.8	*Panaeolus* sp.	1									
BF-OTU563	FR682306	*Psathyrella* cf. *gracilis*	AY228352	91.1	Ag			1							
BF-OTU452	FR682195	*Serpula lacrymans*	AJ536023	99.7	*Serpula lacrymans*	1									
	Cluster 47 - Unknown					1	0	2	0		0	0	1	0	0
BF-OTU655	FR682398	Basidiomycete sp.	AM084842	97.5	Ag								1		
BF-OTU560	FR682303	*Macrotyphula fistulosa*	AJ296348	88.1	Ag §			2							
BF-OTU434	FR682177	Uncultured basidiomycete	AY969416	94.3	Ag	1									
	Cluster 48 - Agaricomycetes					0	0	1	3		5	1	0	1	0
BF-OTU603	FR682346	*Clitocybe nebularis*	DQ486691	99.5	*Clitycybe* sp.									1	
BF-OTU581	FR682324	*Clitocybe subditopoda*	EU669216	98.9	*Clitocybe subditopoda*				1						
BF-OTU680	FR682423	*Clitocybe subditopoda*	DQ202269	99.8	*Clitocybe subditopoda*						1				
BF-OTU660	FR682403	*Clitocybe subditopoda*	EU669216	99.2	*Clitocybe subditopoda*						2				
BF-OTU673	FR682416	*Collybia cirrhata*	AF274382	95.5	Ag						1				
BF-OTU578	FR682321	*Collybia tuberosa*	AF065124	93.6	Ag §				1						
BF-OTU674	FR682417	*Coprinus comatus*	AF345823	91.4	Ag						1				
BF-OTU579	FR682322	*Panellus serotinus*	AY265847	99.3	*Panellus serotinus*				1			1			
BF-OTU525	FR682268	*Tricholoma sejunctum*	AF241518	99.4	*Tricholoma sejunctum*			1							
	Cluster 49 - Agaricomycetes					6	1	7	0		3	2	0	3	0
BF-OTU523	FR682266	*Antrodia sitchensis*	AY966451	99.5	*Antrodia* sp.			6							

Table S1. Detection frequencies and annotation details of NucITS clones and cultivated isolates from house dust samples.

Phylotype name	rDNA tree cluster*/ accession	Nearest Blast hit: Organism	Nearest Blast hit: Accession	Nearest Blast hit: Sim %	Final annotation **	Occurrence in dust and material samples ***: Location 1: In1a	Location 1: Re1a	Location 1: In1b	Location 1: Re1b	Location 1: BM-1	Location 2: In2a	Location 2: Re2a	Location 2: In2b	Location 2: Re2b	Location 2: BM-2
BF-OTU657	FR682400	*Gloeophyllum abietinum*	AJ420947	84.9	Ag						1				
BF-OTU437	FR682180	*Gloeophyllum sepiarium*	AJ420946	100.0	*Gloeophyllum sepiarium*	1									
BF-OTU626	FR682369	*Phlebia radiata*	DQ056859	99.7	*Phlebia radiata*							1		1	
BF-OTU475	FR682218	*Phlebiella vaga*	EU118660	94.9	Ag	1									
BF-OTU664	FR682407	*Pseudotomentella larsenii*	AF326981	92.2	Ag §						1				
BF-OTU482	FR682225	*Resinicium furfuraceum*	DQ873648	98.9	*Resinicium furfuraceum*		1								
BF-OTU701	FR682444	*Steccherinum fimbriatum*	EU118668	99.2	*Steccherinum fimbriatum*							1			
BF-OTU435	FR682178	*Tomentellopsis* sp.	AJ410782	98.5	*Tomentellopsis* sp	1									
BF-OTU441	FR682184	*Trametes ochracea*	AB158314	99.8	*Trametes* sp.	1									
BF-OTU402	FR682145	*Trametes* sp.	AY840569	94.6	Ag	1									
BF-OTU518	FR682261	*Trametes versicolor*	AY309018	99.7	Ag			1							
BF-OTU625	FR682368	*Trechispora alnicola*	DQ411529	86.9	Ag									2	
BF-OTU670	FR682413	Uncultured ectomycorrhiza	AY641458	82.2	Ag						1				
BF-OTU442	FR682185	Uncultured fungus	DQ309195	99.8	Ag §	1									
	Cluster 50 - Unknown					0	0	0	0		1	0	0	0	0
BF-OTU665	FR682408	*Rhizoctonia* sp.	AY927338	72.4	Ag						1				
	Cluster 51 - Unknown					1	0	0	2	1	0	0	0	0	0
BF-OTU466	FR682209	*Bensingtonia* sp.	DQ224375	71.8	Ab	1									
BF-OTU569	FR682312	No full length match			UF				2						
BF-OTU720	FR682462	No match			UF					1					
	Cluster 52 - Microbotryomycetes					0	5	0	0		0	0	1	0	0
BF-OTU490	FR682233	*Occultifur externus*	AF444643	97.5	Cy		1								
BF-OTU509	FR682252	*Rhodotorula lamellibrachiae*	AB263120	86.3	Mi		2								
BF-OTU502	FR682245	*Rhodotorula pinicola*	AF444292	99.8	*Rhodotorula pinicola*		1								
BF-OTU645	FR682388	*Rhodotorula slooffiae*	AF444589	99.8	*Rhodotorula slooffiae*								1		
BF-OTU492	FR682235	*Rhodotorula* sp.	DQ317357	97.6	*Rhodotorula* sp.		1								
	Cluster 53 - Pucciniomycetes					38	0	2	0		3	0	0	0	0
BF-OTU439	FR682182	*Melampsora caprearum*	AY444779	99.0	*Melampsora* sp.	3									
BF-OTU553	FR682296	*Melampsoridium betulinum*	AF125177	100.0	*Melampsoridium betulinum*	7		2							
BF-OTU661	FR682404	*Melampsoridium hiratsukanum*	AY394707	99.8	*Melampsoridium hiratsukanum*	2					1				
BF-OTU428	FR682171	No full length match			UF	1									
BF-OTU424	FR682167	*Thekopsora areolata*	DQ087231	99.8	*Thekopsora areolata*	25					2				
	Cluster 54 - Agaricomycetes					1	0	0	0		0	0	0	0	0
BF-OTU399	FR682142	*Thelephora terrestris*	GQ205371	100.0	*Thelephora terrestris*	1									
	Cluster 55 - Saccharomycetes					0	0	3	0		0	0	0	0	0
BF-OTU538	FR682281	*Galactomyces geotrichum*	AJ279451	99.1	*Galactomyces geotrichum*			1							
BF-OTU547	FR682290	*Galactomyces geotrichum*	DQ148946	100.0	*Galactomyces geotrichum*			1							
BF-OTU566	FR682309	*Yarrowia lipolytica*	DQ683007	86.0	Sa			1							
	Cluster 56 Wallemiomycetes					0	1	0	0		0	0	0	0	0
BF-OTU501	FR682244	*Wallemia muriae*	AY302514	98.8	*Wallemia muriae*		1								
BF036	FR718458	*Wallemia sebi*	AY302517	99.1	*Wallemia sebi*					C					

Table S1. Detection frequencies and annotation details of NucITS clones and cultivated isolates from house dust samples.

Phylotype name	rDNA tree cluster*/ accession	Nearest Blast hit			Final annotation **	Occurrence in dust and material samples ***									
		Organism	Accession	Sim %		Location 1					Location 2				
						In1a	Re1a	In1b	Re1b	BM-1	In2a	Re2a	In2b	Re2b	BM-2
	Cluster 57 - Zygomycetes					1	0	2	0		0	1	0	0	0
BF-OTU548	FR682291	*Mucor hiemalis*	AJ876490	99.8	*Mucor hiemalis*			2							
BF-OTU436	FR682179	*Mucor racemosus*	AY213662	99.8	*Mucor* sp.	1									
BF-OTU685	FR682428	Zygomycete sp.	AY618254	99.3	ZyIs							1			

* rDNA tree cluster: position in the tree shown in Figure 3. The grey area includes the summed frequencies of the OTUs included in the cluster.

** Final annotation: the sequences were annotated by the formerly described method (Ciardo et al. 2007), after which the annotation was refined manually. Phylotypes were annotated to species, genus, class, phylum or as unknown. Abbreviations: Ag: Agaricomycetes; Ab: Agaricostilbomycetes; Art: Arthoniomycetes; AsIs: Ascomycetes *incertae sedis*; BaIs: Basidiomycetes *incertae sedis*; Cy: Cystobasidiomycetes; Do: Dothideomycetes; Eu: Eurotiomycetes; Lc: Lecanoromycetes; Le: Leotiomycetes; Mi: Microbotryomycetes; Pe: Pezizomycetes; Sa: Saccharomycetes; So: Sordariomycetes; Ta: Taphrinomycetes; Tr: Tremellomycetes; UA: unidentified ascomycete; UF: unidentifed fungus; ZyIs: Zygomycetes *incertae sedis.* § An unknown OTU which was either detected in two or more individual libraries or shares high similarity with published environmental sequence.

*** The number of clones belonging to the phylotype in given library is shown. C: the phylotype was cultivated from the sample. The total number of clones in each rDNA tree cluster is shown on the grey field. For building material associated phylotypes, see Table S2 for additional information. Sample name abbreviations: In: index building; Re: reference building; 1: location 1; 2: location 2; a: before-renovation sample; b: after-renovation sample; BM: building material sample pool.
