# Supplementary material for: Molecular profiling of fungal communities in moisture damaged buildings before and after remediation - a comparison of culture-dependent and culture-independent methods
Source: BMC Microbiol. 2011 Oct 21;11:235. doi: 10.1186/1471-2180-11-235 (PMC3206440; doi:10.1186/1471-2180-11-235)
Supplement: Additional file 3 — Table S2: List of fungal phylotypes obtained from building materials by cultivation and clone library analysis. [file 1471-2180-11-235-S3.PDF]

Table S2. List of fungal phylotypes cultivated and cloned from building materials

Phylotype		Phylogenetic affiliation			Incidence			Substrate/ location[f]	qPCR assay[g]
					Index-1		Index-2		
Id.	Accession	Identification[a]	Closest INSD relative (accession no.)	% ID[b]	CL[c]	CU[d]	CU[e]		
		**Dothideomycetes**							
BF048	FR718462	*Pithomyces chartarum* †	*Pithomyces* (*Leptosphaerulina*) *chartarum* (DQ384571)	100.0		2		**M**/B	-
BF060	FR718467	*Cladosporium* sp. *	*Cladosporium herbarum* (AF393712)	100.0		1		n/a	Cherb
BF150	FR718479	*Cladosporium* sp. *	*Cladosporium cladosporioides* (DQ426533)	100.0		1		**W**/R	Cclad2
BF141	FR718476	*Cladosporium* sp. *	*Cladosporium cladosporioides* (AF393691)	100.0			1	**M**/R	Cclad1
BF129	FR718471	*Cladosporium* sp.	*Cladosporium* sp. (AJ300336)	100.0			1	**M**/R	-
BF053	FR718466	*Phoma* sp. † *	*Phoma herbarum* (AY337712)	100.0		1		**M**/B	-
BF153	FR718481	*Phoma herbarum* †	*Phoma herbarum* (AY293800)	100.0			2	**W**/C	-
BF143	FR718477	*Phoma* sp.	*Phoma* sp. (AY465466)	100.0			3	**M**/R	-
BF168	FR718487	*Phoma* sp. †	*Phoma* sp. (AF218789)	100.0			2	**C**/C	-
BF125	FR718470	*Hormonema dematioides* *** †	*Hormonema dematioides* (syn. *Sydowia polyspora*) (AJ278927)	100.0			4	**Ws,M**/R	-
BF-OTU706, BF-OTU707	FR682449, FR682450	UF (Dothideomycetes)	*Mycosphaerella flexuosa* (DQ302958)	83.4	3			n/a	-
		**Eurotiomycetes**							
BF039/ BF-OTU705	FR718460, FR682448	*Phialophora* sp.***	*Phialophora* sp. (AY618679)	99.4	5	2		**W**/W	-
BF029	FR718456	*Paecilomyces variotii*	*Paecilomyces variotii* (AF033395)	100.0		1		**M**/B	Pvari2
BF001	FR718449	*Paecilomyces divaricatus*	*Paecilomyces divaricatus* (AY373851)	100.0		1		**W**/B	-
BF030	FR718457	*Aspergillus sydowii*	*Aspergillus sydowii* (AY373868)	99.6		1		**M**/B	Asydo, Aungu
BF051	FR718464	*Aspergillus nidulans*	*Aspergillus nidulans* (AF455505)	100.0		1		**M**/B	Anidu
BF133	FR718473	*Rhinocladiella atrovirens* †	*Rhinocladiella atrovirens* (AY618683)	98.9			1	**W**/R	-
BF132	FR718472	*Exophiala* sp. *	*Exophiala xenobiotica* (DQ182590)	100.0			5	**Ws, W, M**/R	-
BF043/ BF-OTU704 [§]	FR718462, FR682447	*Aspergillus conicus*	*Aspergillus conicus* (AY373865, EF652039, AY373864)	96.8-99.0	79	1		**W**/ Iw	Arest
BF-OTU648	FR682392	*Eurotium* sp. *	*Eurotium repens* (AY373890)	100.0	1			n/a	Eamst
BF-OTU709-BF-OTU713	FR682452-FR682456	UF (Eurotiomycetes)	*Eupenicillium javanicum* (U18358)	89.0-90.3	52			n/a	-
		**Leotiomycetes**							
BF037/ BF-OTU624	FR718459, FR682367	*Penicillium corylophilum*	*Penicillium corylophilum* (AF034457)	100.0	1	2	2	**W,M**/W, R	-
BF006	FR718452	*Penicillium citreonigrum*	*Penicillium citreonigrum* (AF033456)	100.0		4		**W**/B	-
BF115/ BF-OTU679	FR718468, FR682422	*Penicillium* sp. *	*Penicillium chrysogenum* (AF034451)	99.8	1	3	3	**M,W**/B,R	Pchry, PenGrp2

BF005/ BF-OTU723	FR718451, FR682465	*Penicillium* sp. †	*Penicillium* sp. (FJ379809)	99.1	1	1		**W**/B	-
BF042	FR718461	*Penicillium* sp. *	*Penicillium thomii* (AF034460)	99.8		1		**W**/ Iw	Pspin
BF026	FR718454	*Penicillium* sp.	*Penicillium* sp. (AB297800)	92.0		1		**M**/B	-
BF025	FR718453	*Penicillium* sp. *	*Penicillium commune* (AF236103)	99.8		1		**M**/B	-
BF027	FR718455	*Penicillium* sp. *	*Penicillium canescens* (AF033493)	99.8		1		**M**/B	-
BF167	FR718486	*Penicillium* sp. *	*Penicillium brevicompactum* (AY373898)	100.0			1	**C**/C	Pbrev
BF134	FR718474	UF (Leotiomycetes) **	*Cadophora* sp. (AY371513)	92.8			1	**W**/R	-
		**Sordariomycetes**							
BF002	FR718450	*Thielavia hyalocarpa* †	*Thielavia hyalocarpa* (AB470856)	100.0		1		**W**/B	-
BF-OTU718	FR682460	UF (Sordariomycetes)	*Stilbella byssiseda* (AF335453)	83.5	1			n/a	-
BF052	FR718465	UF (Sordariomycetes)	*Acremonium alternatum* (AM176679)	86.9		1		**M**/B	-
BF124	FR718469	*Trichoderma* sp. *	*Trichoderma citrinoviride* (AJ230663)	100.0			1	**M**/R	Tlong
BF152	FR718480	*Trichoderma* sp. *	*Trichoderma atroviride* (EF417482)	99.8			1	**W**/R	Tviri
BF135	FR718475	*Lecythophora hoffmannii* ***	*Lecythophora hoffmannii* (AY805566)	100.0			2	**W,Ws**/ R	-
		**Microbotryomycetes**							
BF145	FR718478	*Sporidiobolus salmonicolor******	*Sporidiobolus salmonicolor* (AY015434)	100.0			3	**W,M**/R	-
BF165	FR718484	*Rhodotorula mucilaginosa*	*Rhodotorula mucilaginosa* (DQ386306)	100.0			1	**C**/C	-
BF155	FR718482	UF (Microbotryomycetes)	*Rhodosporidium lusitaniae* (AY015430)	94.4			1	**W**/C	-
		**Wallemiomycetes**							
BF036	FR718458	*Wallemia sebi*	*Wallemia sebi* (AY302517)	99.1		1		**M**/B	-
		**Tremellomycetes**							
BF159	FR718483	UF (Tremellomycetes)	*Cryptococcus skinneri* (AF444305)	96.1			1	**Ws**/R	-
BF166	FR718485	*Cryptococcus adeliensis*	*Cryptococcus adeliensis* (AF145328)	100.0			2	**W,C**/C	-
		**Unknown**							
BF-OTU720	FR682462	UF	No Match		1			n/a	-
BF-OTU708	FR682451	UF	No Match		3			n/a	-

a) Identification according to INSD (International Nucleotide Sequence Database) comparisons. Symbols and abbreviations: UF: unidentified fungus,
* The phylotype shared an equal similarity with several INSD sequences, ** Sequence similarity with an uncultivated leaf litter ascomycete (AF502801) 100.0%, *** Isolate was initially identified as *Aureobasidium* by morphology, † Isolate grew as sterile colonies in MEA/DG18 plate culture;
b) Percentage ITS sequence similarity with closest INSD relative;
c) Number of clones belonging to the phylotype in the BM-1 ITS-library constructed from pooled PCR product, see manuscript *Materials and methods*;
d) Number of sequenced isolates cultivated from the building material sample pool BM-1;
e) Number of sequenced isolates cultivated from the building material sample pool BM-2;
f) Source material of isolates (in bold)/source location in building. Abbreviations: **M**: mineral wool, **W**: wood board, **C**: concrete; **Ws**: wind protection lab/B: base floor constructions, R: roof constructions, C: ceiling constructions, W: outer wall constructions; Iw: inner wall constructions. n/a: The source of nucITS clone library OTUs is not known because PCR products from individual samples were pooled prior to cloning.
g) Abbreviation of the EPA qPCR assay(s) used in the study that matches with the phytotype sequence.
§ A cluster of clones (including BF-OTU704, -716, -717, -719, -721, -722, -724 and -552) shared 96.8-99.0% sequence similarities with several INSD reference strains of *Aspergillus conicus*.
