# Supplementary material for: Molecular profiling of fungal communities in moisture damaged buildings before and after remediation - a comparison of culture-dependent and culture-independent methods
Source: BMC Microbiol. 2011 Oct 21;11:235. doi: 10.1186/1471-2180-11-235 (PMC3206440; doi:10.1186/1471-2180-11-235)
Supplement: Additional file 4 — Tables S3 and S4: Concentrations and diversity of fungi determined by culture (S3) and quantitative PCR (S4) in dust. [file 1471-2180-11-235-S4.PDF]

Table S3. Culturable fungal diversity and concentrations in dust ($\log_{10}$(cfu g^{-1})) by location and building.

Genus/group	Location 1				Location 2			
	**In1a**	**In1b**	**Re1a**	**Re1b**	**In2a**	**In2b**	**Re2a**	**Re2b**
Penicillium	4.8	**3.2**	-	-	6.2	6.1	**5.1**	5.4
Yeast	3.0	**3.2**	4.7	-	-	-	3.5	4.0
Cladosporium	4.0	-	6.4	-	-	-	-	-
Aureobasidium	3.7	2.9	-	-	-	-	-	3.7
Acremonium	-	-	5.1	-	-	-	4.1	-
Aspergillus	**2.8**	-	2.7	-	-	-	-	2.7
Unknown	3.7	-	-	-	-	-	-	3.5
Verticillium	3.7	-	-	-	-	-	-	-
Sphaeropsidales	3.6	-	-	-	-	-	-	-
Botrytis	-	-	-	-	-	-	-	**3.0**
Rhizopus	-	-	-	-	-	-	-	2.7
Trichoderma	2.7	-	-	-	-	-	-	-
Alternaria	-	-	-	1.7	-	-	-	-
Eurotium	-	-	-	**1.7**	-	-	-	-
Mucor	1.7	-	-	-	-	-	-	-
Mycelia sterilia	4.0	3.2	-	1.7	-	2.7	3.1	4.0
**TOTAL**	4.9	3.6	6.4	2.0	6.2	6.1	5.1	5.4

The groups are listed according to their total abundance. The higher of the counts yielded from the two media used (MEA and DG18 agar) was included (bold: DG18). For sample name abbreviations, see Table 1.

Table S4. Concentrations of fungi detected by qPCR in dust (log_{10}(CE g^{-1} of dust)) by location and building.

**Assay** [a]	**ERMI cat.** [b]	**Location 1**				**Location 2**			
		**In1a**	**In1b**	**Re1a**	**Re1b**	**In2a**	**In2b**	**Re2a**	**Re2b**
Apull	1	6.9	5.1	7.0	4.8	6.3	5.8	5.8	6.2
Cspha	1	-	-	3.8	-	3.6	2.4	-	2.2
Cclad1	2	5.6	4.4	5.9	4.1	4.8	4.3	4.3	4.8
Cclad2	2	4.7	3.7	5.8	-	4.1	3.1	-	4.1
Cherb	2	6.1	5.2	5.8	4.4	5.7	5.4	4.9	5.8
Enigr	2	4.2	-	3.5	2.4	3.3	3.1	-	2.9
Uatrm		-	-	-	-	4.1	3.6	-	3.4
Afumi	1	-	-	-	5.1	-	4.7	ND	4.0
Apeni2	1	-	-	-	-	6.3	5.2	-	6.1
Arest	1	-	-	-	-	-	3.8	-	4.9
Eamst	1	5.4	4.6	5.0	3.9	5.1	4.3	4.6	4.6
Acaes		-	-	-	-	-	2.7	-	-
Acand3		-	-	-	-	-	2.7	-	2.7
Anigr		-	-	-	-	-	3.6	-	3.8
Apard		-	4.8	-	-	4.6	-	-	-
Tviri	1	5.2	-	-	-	4.1	4.0	-	3.4
Tharz		-	-	-	-	-	-	4.6	3.1
Tlong		-	-	-	-	-	-	6.5	-
Pbrev	1	6.4	-	-	-	-	4.6	-	4.7
Pvarb	1	4.7	-	-	-	-	-	-	-
PenGrp2	1	-	-	-	-	5.8	ND	-	ND
Pchry	2	-	-	-	-	6.8	6.2	6.8	5.0
Pdigi		-	-	-	-	4.0	3.1	-	-
Pedecu2-5x		-	-	-	4.9	-	-	7.3	-
Polsn		-	-	-	-	-	-	5.1	4.8
Proqu		-	-	-	-	-	-	-	3.5
PenGrp1		-	-	-	-	-	4.3	-	-
SCbrv	1	-	-	-	-	-	-	-	2.5
SCchr	1	-	-	-	3.5	-	-	-	2.7
Astrc	2	-	-	-	-	-	3.8	-	4.4
Wsebi	1	4.8	-	-	-	4.4	3.9	4.3	4.5
Muc1	2	4.7	-	5.2	4.8	3.5	3.2	-	3.4
Rstol	2	4.0	-	3.8	-	3.0	-	-	3.3
**Total CE in sample**		7.1	5.6	7.1	5.6	7.1	6.4	7.5	6.6
**ERMI in sample**		4.0	-0.7	-5.2	1.0	4.4	4.3	-1.3	4.6

The assays are listed by genus according to their total abundance. [a] A full list of performed qPCR assays with included species are listed in Table S6. [b] The assays marked with "1" and "2" are used to calculate the ERMI values; category 1 species are considered as moisture damage associated fungi, and increase the ERMI value while species marked with 2 belong to common airborne fungi and decrease the ERMI value. For sample name abbreviations, see Table 1.
