# Supplementary material for: Molecular profiling of fungal communities in moisture damaged buildings before and after remediation - a comparison of culture-dependent and culture-independent methods
Source: BMC Microbiol. 2011 Oct 21;11:235. doi: 10.1186/1471-2180-11-235 (PMC3206440; doi:10.1186/1471-2180-11-235)
Supplement: Additional file 5 — Fig. S2: Comparison of clone library frequencies and qPCR cell counts for fungal phylotypes targeted by mold specific qPCR. [file 1471-2180-11-235-S5.PDF]

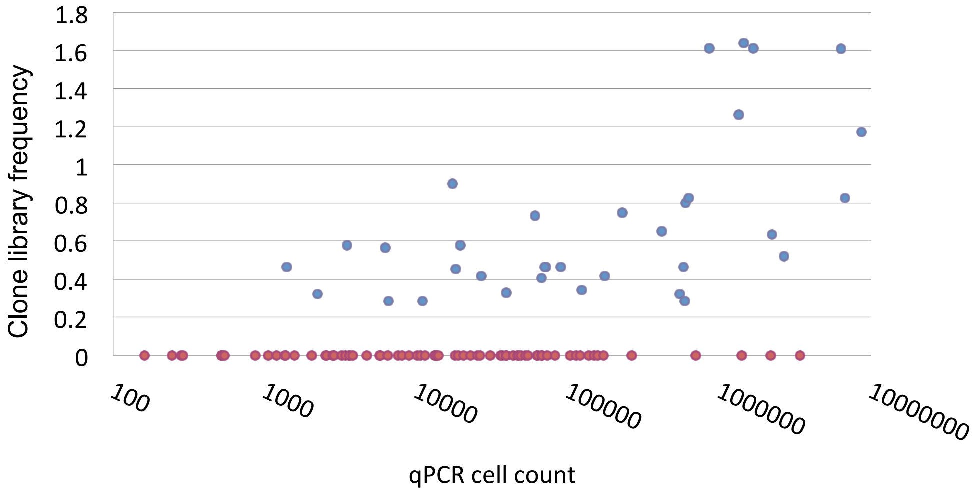

Fig. S2. A dot-plot showing log(10) transformed values of clone library frequencies and qPCR cell counts for fungal phylotypes targeted by mold specific qPCR. Phylotypes detected by both clone library sequencing and qPCR method are shown in blue, while phylotypes detected solely by qPCR are shown in red.
