# Supplementary material for: Molecular profiling of fungal communities in moisture damaged buildings before and after remediation - a comparison of culture-dependent and culture-independent methods
Source: BMC Microbiol. 2011 Oct 21;11:235. doi: 10.1186/1471-2180-11-235 (PMC3206440; doi:10.1186/1471-2180-11-235)
Supplement: Additional file 6 — Table S5: Statistical pair-wise comparison of nucITS clone libraries from settled dust samples. [file 1471-2180-11-235-S6.PDF]

Table S5. Statistical pair-wise comparison of ITS clone libraries from settled dust samples

Compared samples	Sørensen similarity		Unifrac
	QS [a]	Ab-CS [b]	distance D [c]
**1st sampling**			
In1a-Re1a	0.20	0.51	0.754**
In1a-In2a	0.21	0.27	0.685
Re1a-Re2a	0.09	0.07	0.753**
In2a-Re2a	0.14	0.23	0.654*
Mean (all pairs) [e]	0.15	0.23	
**2nd sampling**			
In1b-Re1b	0.07	0.16	0.802**
In1b-In2b	0.20	0.36	0.751**
Re1b-Re2b	0.13	0.48	0.714**
In2b-Re2b	0.19	0.98	0.692*
Mean (all pairs) [e]	0.15	0.42	
**Location 1**			
In1a-Re1a	0.20	0.51	0.754**
In1a-In1b	0.23	0.34	0.645
Re1a-Re1b[d]	0.08	0.09	0.738
In1b-Re1b	0.07	0.16	0.802**
**Location 2**			
In2a-Re2a	0.14	0.23	0.654*
In2a-In2b	0.16	0.61	0.715*
Re2a-Re2b	0.25	0.71	0.606*
In2b-Re2b	0.19	0.98	0.692*

[a)] Classic Sørensen index for pairwise similarity of observed OTU content; [b)] Chao's abundancy based Sørensen index for pairwise similarity of estimated OTU content. Sørensen indices were calculated from a random sample of 100 clones from each library apart from the sample Re1b from which only 26 clone sequences were obtained and used; [c)] D: tree-based Unifrac distance calculated from sequence data without abundance weighting; *Suggestively significant pairwise difference, **significant pairwise difference (Unifrac significance test); [d)] the low clone number of library Re1b (26 sequences) may have biased the results. [e)] Mean value of all pair-wise comparisons of the 4 buildings.
