# Supplementary material for: Molecular profiling of fungal communities in moisture damaged buildings before and after remediation - a comparison of culture-dependent and culture-independent methods
Source: BMC Microbiol. 2011 Oct 21;11:235. doi: 10.1186/1471-2180-11-235 (PMC3206440; doi:10.1186/1471-2180-11-235)
Supplement: Additional file 7 — Table S6: List of performed qPCR assays and targeted species. [file 1471-2180-11-235-S7.PDF]

Table S6. List of performed qPCR assays and targeted species
(source: http://www.epa.gov/nerlcwww/moldtech.htm accessed March 2011)

**Assay name**	**Target species**
Aaltr	*Alternaria alternata*
Acaes	*Aspergillus caespitosus*
Acand3	*Aspergillus candidus*
Acerv	*Aspergillus cervinus*
Aclav	*Aspergillus clavatus, A. giganteus*
Aflav	*Aspergillus flavus, A. oryzae*
Aflp2	*Aspergillus flavipes*
Afumi	*Aspergillus fumigatus, Neosartorya fischeri*
Anidu2	*Emericella* (*Aspergillus*) *nidulans, E. rugulosa, E. quadrilineata*
Anigr	*Aspergillus niger, A. awamori, A. foetidus, A. phoenicis*
Anive	*Aspergillus niveus*
Aochr1	*Aspergillus ochraceus, A. ostianus*
Apard	*Aspergillus paradoxus*
Apeni2	*Aspergillus penicillioides*
Apull	*Aureobasidium pullulans*
Arest	*Aspergillus restrictus, A. caesillus, A. conicus*
Asclr	*Aspergillus sclerotiorum*
Astrc	*Acremonium strictum*
Asydo3	*Aspergillus sydowii*
Atama2	*Aspergillus tamarii*
Aterr2	*Aspergillus terreus*
Aungu	*Aspergillus unguis*
Austs2	*Aspergillus ustus*
Avari	*Emericella (Aspergillus) variecolor*
Awent	*Aspergillus wentii*
Avers2-2	*Aspergillus versicolor*
Cclad1	*Cladosporium cladosporioides*, svar. 1
Cclad2	*Cladosporium cladosporioides*, svar. 2
Cglob	*Chaetomium globosum*
Cherb	*Cladosporium herbarum*
Cspha	*Cladosporium sphaerospermum*
Eamst	*Eurotium (Aspergillus) amstelodami, E. chevalieri, E. herbariorum, E. rubrum, E. repens*
Enigr	*Epicoccum nigrum*
Mem	*Memnoniella echinata*
Muc1	*Mucor amphibiorum, M. circinelloides, M. hiemalis, M. indicus, M. mucedo, M. racemosus, M. ramosissimus, Rhizopus azygosporus, R. homothalicus, R. microsporus, R. oligosporus, R. oryzae*
Patra	*Penicillium atramentosum*
Pbrev	*Penicillium brevicompactum, P. stoloniferum*
Pchry	*Penicillium chrysogenum* svar. 2
Pcitr	*Penicillium citrinum, P. sartoryi, P. westlingi*
Pcory	*Penicillium corylophilum*
Pdigi	*Penicillium digitatum*
Pdecu3	*Penicillium decumbens*
PenGrp1	*Penicillium aurantiogriseum, P. freii, P. hirsutum, P. polonicum, P.*

	tricolour, P. viridicatum, P. verrucosum svar. 2
PenGrp2	*Penicillium crustosum, P. camembertii, P. commune, P. echinulatum, P. solitum*
Pimpl	*Penicillium implicatum*
Pisla	*Penicillium islandicum*
Polsn	*Penicillium olsonii*
Poxal	*Penicillium oxalicum*
Ppurp	*Penicillium purpurogenum*
Prais3	*Penicillium raistrickii*
Prest2	*Penicillium restrictum*
Proqu	*Penicillium roquefortii*
Psimp2	*Penicillium simplicissimum, P. ochrochloron*
Psclr	*Penicillium sclerotiorum*
Pspin2	*Penicillium glabrum, P. lividum, P. purpurescens, P. spinulosum, P. thomii*
Pvarb2	*Penicillium variabile*
Pvari2	*Paecilomyces variotii*
Rstol	*Rhizopus stolonifer*
SCbrv	*Scopulariopsis brevicaulis, S. fusca*
SCchr	*Scopulariopsis chartarum*
Stac	*Stachybotrys chartarum*
Taspr1	*Trichoderma asperellum, T. hamatum*
Tharz	*Trichoderma harzianum*
Tlong	*Trichoderma longibrachiatum, T. citrinoviride*
Tviri	*Trichoderma viride, T. atroviride, T. koningii*
Uatrm	*Ulocladium atrum*
Ubotr	*Ulocladium botrytis*
Uchar	*Ulocladium chartarum*
Wsebi	*Wallemia sebi*
