# Supplementary material for: Molecular profiling of fungal communities in moisture damaged buildings before and after remediation - a comparison of culture-dependent and culture-independent methods
Source: BMC Microbiol. 2011 Oct 21;11:235. doi: 10.1186/1471-2180-11-235 (PMC3206440; doi:10.1186/1471-2180-11-235)
Supplement: Additional file 8 — Table S7: Summary of analysed samples and applied methods. [file 1471-2180-11-235-S8.PDF]

Table S7. Summary of the analysed samples and applied methods.

Sample information				Analyses			
Sample id.	Building[a]	Sampling time	Sample type[b]	nucITS clone library	qPCR	Culture[c]	Erg[d]
In1a	Index-1	Feb.-Mar. 2003 (before renovation)	D	+	+	VC	+
In1b	Index-1	Feb.-Mar. 2004 (after renovation)	D	+	+	VC	+
Re1a	Reference-1	Feb. 2003	D	+	+	VC	+
Re1b	Reference-1	Feb.-Mar. 2004	D	+	+	VC	+
BM-1*	Index-1	during renovation	BM	+	-	S	-
In2a	Index-2	Mar.-Apr. 2004 (before renovation)	D	+	+	VC	+
In2b	Index-2	Feb.-Apr. 2005 (after renovation)	D	+	+	VC	+
Re2a	Reference-2	Mar.-Apr. 2004	D	+	+	VC	+
Re2b	Reference-2	Feb.-Apr. 2005	D	+	+	VC	+
BM-2*	Index-2	during renovation	BM	-	-	S	-

a) All studied buildings are located in central Finland, Europe (62-64° N); b) Abbreviations: D: settled dust from above-floor surfaces; BM: building material samples; c) Abbreviations: VC: viable counting and genus-level identification of isolates by morphology; S: identification of isolates by morphology and nucITS-sequencing. d) Measurement of the ergosterol concentration. * BM-1: pool of building material samples collected from the Index-1 building during renovation, n=7; * BM-2: pool of building material samples collected from the Index-2 building, n=9.
